# Physical, but not chemical, antiherbivore defense expression is related to the clustered spatial distribution of tropical trees in an Amazonian forest

**DOI:** 10.1002/ece3.4859

**Published:** 2019-01-31

**Authors:** Johanna Cobo‐Quinche, María‐José Endara, Renato Valencia, Dolly Muñoz‐Upegui, Rafael E. Cárdenas

**Affiliations:** ^1^ Herbario QCA, Laboratorio de Ecología de Plantas, Escuela de Ciencias Biológicas, Facultad de Ciencias Exactas y Naturales Pontificia Universidad Católica del Ecuador Quito Ecuador; ^2^ Department of Biology University of Utah Salt Lake City Utah; ^3^ Centro de Investigación de la Biodiversidad y Cambio Climático Universidad Tecnológica Indoamérica Quito Ecuador; ^4^ Museo de Zoología QCAZ, Laboratorio de Entomología, Escuela de Ciencias Biológicas, Facultad de Ciencias Exactas y Naturales Pontificia Universidad Católica del Ecuador Quito Ecuador

**Keywords:** conspecific negative density dependence, damage leaf resistance, Ecuador, metabolomics, monodominance, ontogeny, physicochemical leaf traits, Yasuní National Park

## Abstract

1. The conspecific negative density dependence hypothesis states that mortality of young trees (seedlings and saplings) is higher near conspecific adults due to mechanisms such as allelopathy, intraspecific competition, and pest facilitation, explaining why in the tropics, most of plant species tend to be rare and live dispersed. However, there are some tree species that defy this expectation and grow in large clusters of conspecific juveniles and adults. We hypothesize that conspecifics living in clusters show higher and/or more variable defensive profiles than conspecifics with dispersed distributions.
2. We evaluated our hypothesis by assessing the expression of physical leaf traits (thickness, and the resistance to punch, tear and shear) and leaf chemical defenses for six clustered and six non‐clustered tree species in Yasuní National Park, Ecuadorian Amazon. We ask ourselves whether (a) clustered species have leaves with higher physical resistance to damage and more chemical defenses variability than non‐clustered species; (b) saplings of clustered species may show higher physical resistance to damage and higher variation on chemical leaf defenses than their conspecific adults, and (c) saplings of non‐clustered species show lower resistance to physical damage and lower variation in chemical defenses compared to conspecific adults.
3. Overall, our study did not support any of our hypotheses. Remarkably, we found that soluble metabolites were significantly species‐specific.
4. Our study suggests that plants physical but not chemical leaf antiherbivore defenses may be a crucial strategy for explaining survivorship of clustered species. Trees in Yasuní may also fall along a suite of tolerance/escape/defense strategies based on limitations of each species physiological constraints for survival and establishment. We conclude that other mechanisms, such as those related to indirect defenses, soil nutrient exploitation efficiency, volatile organic compounds, delayed leaf‐greening, and seed dispersal mechanisms, shall be evaluated to understand conspecific coexistence in this forest.

## 1 INTRODUCTION

High diversity of plant species in the tropics is hypothesized to be maintained through negative density‐dependent interactions among species and their specialized natural enemies (Bagchi et al., 2010; Connell, 1971; Janzen, 1970). When seedlings and saplings near conspecific adults are more likely to die due to pest facilitation or competition than seedlings and saplings of non‐clustered or rare species (e.g., with <3 individuals/ha), it results in what has been called conspecific negative density dependence (CNDD; Johnson, Beaulieu, Bever, & Clay, 2012; LaManna et al., 2017; Wright, 2002).

**Figure 1:**
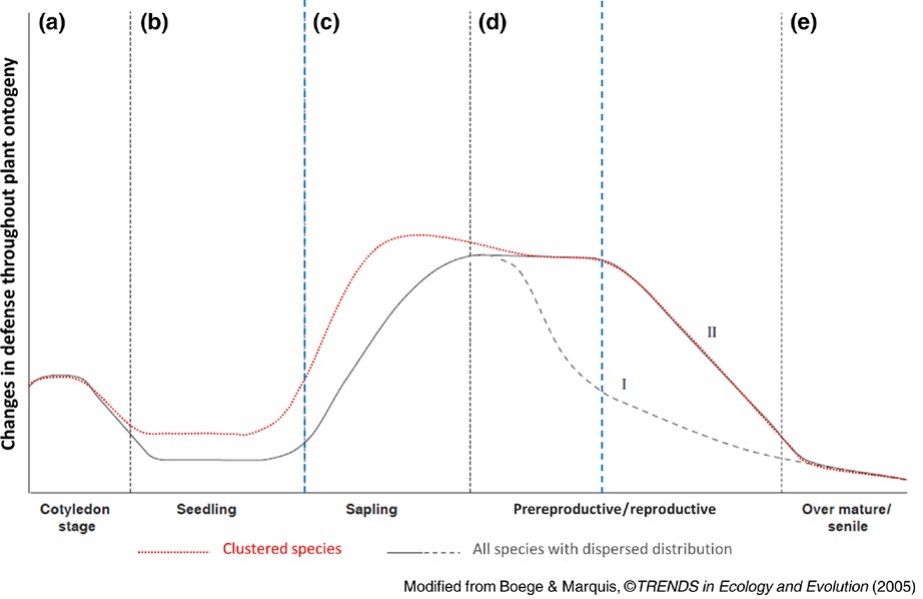
Hypothesized patterns of defense expressions during plant ontogeny of species growing in clusters (red dotted line) and species with dispersed distribution (gray full and dashed lines). Blue vertical dashed lines represent the ontogenetic stages (saplings and adults) considered in this study. According to the 95th percentile of the YFDP data, individuals with dbh < 65 mm were considered as saplings, and individuals with a dbh < 310 mm were considered as adults. Boege and Marquis (2005) suggest that saplings (c) would be more vulnerable to pathogen outbreaks, whereas mature plants (d) in non‐reproductive stages would reach their maximum defensive or tolerance rate to herbivore attack. Decrease in phases (I) or (II) shows reproductive stage or plant senescence, respectively. The red dotted line represents our proposed pattern of defense for clustered species, where saplings (c) would be better defended against herbivory than their adult counterparts (d) in order to survive. Figure modified from Boege and Marquis (2005), with permission of *TRENDS in Ecology & Evolution*

Numerous studies have documented the effects of CNDD in species‐rich tropical forests both including seeds and seedling mortality (Chen, Umaña, Uriarte, & Yu, 2018; He & Duncan, 2000; Johnson et al., 2012; LaManna et al., 2017; Metz, Souza, & Valencia, 2010; Peters, 2003; Wright, 2002), and sapling survival as well (Peters, 2003; Ramage et al., 2017). However, there are some common tree species (e.g., >50 individuals/ha) that grow in large clusters of conspecific saplings and adults, defying the expectations of the CNDD. There are particular instances when some tropical tree species can out‐compete others and become monodominant or live in clusters in a given area, for example, the lack of exogenous disturbances over long periods where competitively superior species are best adapted to particular environmental conditions (Peh, Lewis, & Lloyd, 2011). Or species‐specific life‐history traits such as the type of seed dispersal, which is generally over short distances (e.g., ballistic type of dispersion; Bagchi et al., 2011; Lopes, 2007; Peh et al., 2011; Silva & Souza‐Lima, 2013), may favor the establishment of large numbers of conspecifics in a given area at the expense of other species (Corrales, Mangan, Turner, & Dalling, 2016; Peh et al., 2011). A potential mechanism for survival of young trees of clustered species could be by shifts in the expression of leaf traits associated with antiherbivore defenses to avoid CNDD. Unlike Boege and Marquis (2005) theoretical proposal (see Figure 1), clustered species in the tropics may allocate resources primarily assigned to defense in non‐reproductive stages, allowing them to face herbivory, grow healthy, and reach adulthood (Figure 1; Boege, Barton, & Dirzo, 2011; Smith, Want, O'Maille, Abagyan, & Siuzdak, 2006). For this, saplings of clustered species may show greater values involving defenses and higher variation in their expression than adult conspecifics. Hence, such condition would not share enemies with their neighbors, leading to fitness advantages to survive and coexist. Sánchez‐Hidalgo, Martínez‐Ramos, and Espinosa‐García (1999) found that chemistry of seedlings (leaf terpenoids) under adult conspecifics differs significantly from the latter, explaining how they could survive in cluster conditions supporting the Langeheim and Stubblebine (1983) hypothesis.

Here, we evaluate if the divergence of defensive leaf traits of trees explains the high density and clustering of some species in a hyperdiverse Amazonian forest. Accordingly, on twelve tree species with extreme spatial‐type distributions (six clustered vs. six non‐clustered species) in the hyperdiverse Yasuní forest, Ecuadorian Amazon, we analyzed physical and chemical defenses, including leaf‐toughness traits (Onoda et al., 2011) and secondary metabolites (Patti, Yanes, & Siuzdak, 2013), which have been proved to have a defensive role against herbivory (Cárdenas, Valencia, Kraft, Argoti, & Dangles, 2014; Moles et al., 2013; Pérez‐Harguindeguy et al., 2013). Specifically, we ask whether (a) clustered species have leaves with higher physical resistance to damage and more chemical defenses variability than non‐clustered species. Based on an ecological theory (Boege & Marquis, 2005), (b) saplings of clustered species may show higher physical resistance to damage and higher variability on chemical leaf defenses than their conspecific adults, and (c) saplings of non‐clustered species may show lower resistance to physical damage and lower variability in chemical defenses compared to conspecific adults.

## 2 MATERIALS AND METHODS

### 2.1 Study area

The Yasuní National Park (YNP) and the adjacent Waorani Indigenous territory together cover 1.6 million ha. These territories form the largest protected area in the Amazonian Ecuador (~17.7% of the Ecuadorian Territory; Valencia, Foster, et al., 2004, Supporting Information Appendix S1) and harbor one of the world's most diverse tropical forests (Bass et al., 2010). The YNP is an evergreen lowland wet forest ranging in altitude from 200 to 300 m above sea level, characterized by 15–30 m of canopy with some emergent trees reaching 50 m (Valencia, Condit, et al., 2004). Rainfall and temperature are aseasonal, with a mean annual rainfall of 2,826 mm (none of the 12 calendar months averaging <100 mm) and a mean monthly temperature ranging from 22 to 32°C (min: 16.9; max: 38.9°C; see Valencia, Foster, et al., 2004 for more details; data obtained from Yasuní Research Station).

This study was conducted at the “Yasuní Forest Dynamic Plot” (YFDP), located at the south of the Yasuní Research Station of the *Pontificia* *Universidad Católica del Ecuador* (YRS‐PUCE; 76°24′1.8″W; 00°40′16.7″S). The YFDP is a 50‐ha. plot (500 × 1,000 m) created in 1995 and established as part of a global network of permanent forest dynamic plots (76°23′72″W; 00°41′14″S, coordinates corresponding to the SW corner of the plot; see http://www.puce.edu.ec/portal/content/Dinamica del Bosque Yasuní and Supporting Information Appendix S1 for more details). Repeated censuses since 1995 make possible to monitor the long‐term demography of thousands of plant species within the plot and explain their dynamics with ecological theories (Valencia, Foster, et al., 2004).

### 2.2 Species selection

Prior to choosing the plants species for this study, we calculated the degree of spatial clustering of individuals in the plot of about 100 common species using scalewise variances and moment equations, such as the spatial distribution variance as a function of spatial scale calculated with wavelet kernel functions as developed by Detto and Muller‐Landau (2013). This procedure determinates the probability distribution of independently observed scalewise variances for a given expectation, including complete spatial randomness. This technique provides an analytical test of the null model of spatial randomness to understand at which scale, if any, the variance departs significantly from randomness, resulting in a given wavelet variance value (Detto & Muller‐Landau, 2013). The test clearly identified 12 common species (those with >1,000 individuals in all 50‐ha. plot) that showed contrasting spatial patterns (Table 1). Six species showed wavelet variances ≥33.28 denoting they are spatially clustered and six species showed wavelet variances ≤9.39 representing plants with dispersed distributions.

**Table 1:** List of plant species, and taxonomical position and type of seed dispersal and dispersal mechanism based on specialized literature

Species name	Family	Order	Type of clustering status	Type of seed dispersal	Dispersal mechanism	Ant presence	Young leaf color*	Code
*Acalypha cuneata* Poepp.	Euphorbiaceae	Malpighiales	clustered	autochory	ballistic	No	NG	Ac
*Acidoton nicaraguensis *(Hemsl.) G. L. Webster	Euphorbiaceae	Malpighiales	clustered	autochory	ballistic	No	NG	An
*Rinorea apiculata* Hekking	Violaceae	Malpighiales	clustered	autochory	ballistic	No	NG	Ra
*Rinorea viridifolia *Rusby	Violaceae	Malpighiales	clustered	autochory	ballistic	No	NG	Rv
*Macrolobium *“yasuni” [nomen nudum]	Fabaceae	Fabales	clustered	autochory	ballistic	No	R	My
*Matisia oblongifolia *Poepp. & Endl.	Malvaceae	Malvales	clustered	allochory	animals	No	NG	Mo
*Pourouma bicolor*Mart.	Urticaceae	Rosales	non‐clustered	allochory	animals	Yes	P	Pb
*Sorocea steinbachii *C. C. Berg	Moraceae	Rosales	non‐clustered	allochory	animals	No	NG	Ss
*Eugenia *“minicomun” [nomen nudum]	Myrtaceae	Myrtales	non‐clustered	allochory	animals	No	NG	Em
*Matisia malacocalyx *(A. Robyns & S. Nilsson) W. S. Alverson	Malvaceae	Malvales	non‐clustered	allochory	animals	No	NG	Mm
*Neea *“comun” [nomen nudum]	Nyctaginaceae	Caryophyllales	non‐clustered	allochory	animals	No	NG	Nc
*Eschweilera coriacea* (DC.) S. A. Mori	Lecythidaceae	Ericales	non‐clustered	allochory/autochory	animals‐wind/ballistic	No	NG	Ec

^*^Young leaf color was placed in the following color categories: normally greening (NG), reddish (R), pale (P; i.e., delayed greening).

Using 2D Kernel density estimation analysis, we obtained a map of spatial density interpolation to identify the sites of higher density of individuals of the 12 common species. We chose five different sites per species (60 sites in total) where clustered and non‐clustered species (including adults and saplings) were most densely distributed. A list of the plant species used in this study with further taxonomic, reproductive strategies, and species information are shown in Table 1 (based on Basset, 2001; Bagchi et al., 2011; Lopes, 2007;Pérez, Hernández, Romero‐Saltos, & Valencia, 2014; Queenborough, Metz, Valencia, & Wright, 2013; Silva & Souza‐Lima, 2013).

### 2.3 Leaf sampling

In the field, we verified that each site fitted into a uniform understory habitat, avoiding any gap, and held a considerable number of healthy young and adult trees in the surroundings—that is, individuals visually showing <20% of damaged leaves by herbivores or pathogen fungi and/or bacteria. We also were sure our chosen trees were in non‐reproductive stages (flowering or fructification). Based on the YFDP census data (Valencia, Condit, et al., 2004; Valencia *et al*., unpublished), we used the 5th and 95th percentiles of the diameter at the breast height (dbh) of targeted individuals of each of our focal species to help define whether they belonged to sapling or adult stages (Supporting Information Table S1 in Appendix S2). The sapling stage is a vulnerable life stage in recruitment from seedling to adult because it can persist for decades due to slow growth in the low light of shaded understory. Long‐term data from Barro Colorado Island (BCI), Panama, showed, for example, that the median height of saplings after 20 years of growth was just 38 cm (Wright, 2002). Thus, during this life stage, deterministic diversifying processes have long‐lasting impacts on the composition and relative abundance of species in tropical forests (Green, Harms, & Connell, 2014). We affirmed that all the detailed information we had in hand from the plot that those categorized specimens as “saplings” were new recruits; hence, we avoided any possibility to work with plants that have entered the mature stage before they actually start flowering (Jones, 1999). Because most herbivore attacks take place when leaves are not fully expanded yet, we collected only from the understory those, expanded leaves not so far from “age zero”—that is, just when leaves have expanded and reached their full size but are still clearly new (such foliage typically show bright green colors, lustrous and are softer to touch; Cárdenas et al., 2015; Kursar & Coley, 2003). At this stage, leaves are still palatable for herbivores because they are not completely tough (Coley & Kursar, 2014; Kursar & Coley, 2003). Between 4 and 10 recently expanded leaves were collected from each sampled individual, except from *Eugenia “*minicomun,” a species that presents relatively small leaves (average of 20 leaves/individual were collected). From each lot of collected leaves, we randomly chose two leaves for physical leaf‐trait characterization (eight leaves for *E*. “minicomun”) while remaining leaves were used for chemical analyses.

### 2.4 Physical leaf‐trait analyses

Physical analyses included leaf thickness and leaf resistance to tear, punch, and shear. Leaf thickness plays a key role in determining the physical strength of leaves (Niinemets, 1999; Pérez‐Harguindeguy et al., 2013), whereas the other leaf resistance traits have shown to be negatively related to herbivore attack (Cárdenas et al., 2014; Carmona, Lajeunesse, & Johnson, 2011; Cornelissen et al., 2003; Moles et al., 2013). After collection, foliar material was constantly hydrated using an atomizer and rain water to maintain natural levels of cell turgor (Cornelissen et al., 2003). Leaf lamina thickness was measured using an analogue 0–25 mm micrometer caliper (0.005 mm precision, Amico Corporation, Ontario, Canada), avoiding primary and secondary veins. All physical analyses were performed following the methodology of Cárdenas et al. (2014).

To measure leaf resistance, we used a digital dual range force sensor (Vernier Software & Technology, Beaverton, Oregon, USA). This sensor was fixed to a handmade steel instrument that allowed standard movements for all the tests. Forces were measured in Newtons (N) at 0.01 N precision. When leaves exhibited resistances >10 N, resistance force was measured at 0.05 N precision as suggested by the manufacturer. Only leaves of *Pourouma bicolor* and *Neea* “comun” were measured at 0.05 N precision.

For tearing tests, we cut a leaf segment from the central part of the leaf, in parallel to its main axis and avoiding the midrib. The leaf strip was fixed between two underpressure plaques—one fixed, the other mobile—leaving a space for tearing. The force sensor was attached to an arm that performed a horizontal movement. A rubber bumper screwed directly to the sensor pushed the mobile plaque until the leaf strip was ripped. The tearing tests measured the maximum forces to tear (N_max_; Onoda et al., 2011).

For the punching tests, we screwed on the force sensor a 1.68‐mm‐diameter rod of aluminum flat‐ended rivet (tip area: 2.22 mm^2^) to perpendicularly punch the leaves, avoiding primary and secondary veins. Measurements were recorded as the maximum force per unit of fracture (N_max_ × mm^−2^; Onoda et al., 2011).

For the shearing tests, we screwed to the force sensor a peg‐like folded steel sheet that held a razor blade on the force sensor (Procter & Gamble Co., Río de Janeiro, Brazil). The leaf was fixed between two pressure plates, leaving a space for shearing. Two perpendicular movements were made to cut the leaves at ¼ from the bottom and ¾ from the tip of each leaf. Measurements were recorded as the force per unit of time (s × N; the area under the curve) and normalized to the force per unit of fracture (N × s × mm^−1^; Onoda et al., 2011).

### 2.5 Chemical leaf‐trait analyses

In the field, the remaining expanded leaf samples were dried at 45°C for 48–72 hr and transported to the University of Utah for chemical analyses. In Utah, samples were homogenized using a ball mill grinder at 30 Hz for 40 s (MM 200; Retsch, Haan, Germany) and kept in dry conditions. The methodology described below is specific for extracting secondary soluble metabolites (no primary metabolites were extracted nor analyzed). Those compounds covalently bound to the cell wall were also excluded (Endara et al., 2015; Wiggins, Forrister, Endara, Coley, & Kursar, 2016). Preliminary chromatographic analyses were performed to detect the optimal number of samples required for chemical analyses (Supporting Information Appendix S3). Approximately, 0.0975–0.1025 g of homogenized sample was mixed with 1.0 ml of extraction buffer (60% acetate and 40% acetonitrile). After extraction for 10 min and centrifugation (18,928 *g*) for 5 min, the supernatant was transferred to a newly labeled tube. This procedure was repeated for two times. After removal of the supernatant, the pellet was vacuum‐dried and weighed to quantify total chemical investment.

For the chromatographic analyses, 200 µl of supernatant with 1 µl of 40 mg/ml of cortisol in acetonitrile was transferred to a HPLC vials for new sample concentrations. The samples were analyzed as “undiluted” because peaks were not well visible in diluted concentrations. From these samples, soluble metabolites were analyzed using ultra performance liquid chromatography and mass spectrometry (UPLC‐MS) with an Acquity UPLC^®^ *I*‐*Class* system and a Xevo^®^ G2 Q‐ToF MS equipped with LockSpay^TM^ (Waters, Milford, MA; Further details of UPLC‐MS parameters are in Supporting Information Appendix S2).

Raw data from the UPLC/MS were processed from spectroscopic peaks for detection of retention times and m/z ranges during peak alignment, using XCMS (Smith et al., 2006). Data filtering was performed using the interquartile range. Peak intensities or total ion current was normalized using normalization by sum, and data scaling was performed using autoscaling in the MetaboAnalyst web server 3.0 (Xia & Wishart, 2016).

### 2.6 Statistics

#### 2.6.1 Physicochemical analyses: clustered versus non‐clustered species

To evaluate the type of data distribution, we analyzed the distribution of residuals using Shapiro–Wilk normality tests. Because we found no normal distributions of residuals (Supporting Information Table S1 in Appendix S4), differences in physical leaf traits and chemical investment between clustered and non‐clustered were analyzed using Mann–Whitney test. To test chemical defenses variability, differences in coefficients of variation (CV) were assessed by asymptotic test (Feltz & Miller, 1996; Krishnamoorthy & Lee, 2014) using the library “cvequality” in the R software (R Development Core Team, 2015). We also ran a PCA analysis to determine the multivariate relationships for all the suite of physical leaf traits. Except for CV, analyses were run in PAST statistical software v3.07 (Hammer, Harper, & Ryan, 2001).

#### 2.6.2 Physical analyses: Saplings versus adult trees

We first evaluated the normality distribution of residuals using Shapiro–Wilk normality tests. Differences in physical leaf traits between saplings and adults for all the pool of clustered, non‐clustered, and all species were evaluated by *t* tests since we found normal distributions of residuals (Supporting Information Table S1 in Appendix S5). Analyses were performed in PAST statistical software v3.07 (Hammer et al., 2001).

#### 2.6.3 Differences in leaf chemistry among saplings and adult trees

Investment in chemical defenses was estimated gravimetrically. Differences between saplings and adults were evaluated using *t* tests after confirming normal distribution of residuals using Shapiro Wilk tests (Supporting Information Table S1 in Appendix S6). Analyses were run in PAST statistical software v3.07 (Hammer et al., 2001).

To quantify metabolome‐wide variation among treatments, we used multivariate statistical methods. These analyses were performed in the MetaboAnalyst web server 3.0 (Xia & Wishart, 2016). We used heatmaps to visualize differences on the presence and absence of compounds across ontogenetic stages. A PCA model, a PLS‐DA model, and a hierarchical clustering analysis were fitted to the data in order to analyze grouping patterns. When a PCA was not significant, PLS‐DA and hierarchical clustering were not performed. Validity of group assignments was assessed in the PLS‐DA using a statistical test of permutation based on the separation distance between‐group sum of the squares and within‐group sum of the squares (B/W ratio; Xia & Wishart, 2016).

#### 2.6.4 Effect of the level of the spatial clustering of species on physicochemical traits at ontogenetic stages

Using simple linear and non‐linear regression models (e.g., logarithmic, hyperbolic, exponential), we evaluated the relationships between the level of spatial clustering for variation of physicochemical traits and for physical leaf traits for the 12 common species. Regressions were performed using Table Curve^®^ 2D v5.01 (Systat Software, Inc., Richmond, California, USA) where ANOVA statistical values from each fitted equation were obtained.

#### 2.6.5 Neighborhood effect on physical traits

For every individual of the 12 common species, we used Pearson correlation analyses to determine the relationship between physical leaf traits and the level of spatial clustering (wavelet variance) as a function of the distance between surrounding conspecifics at the whole 50‐ha plot level. For this, we calculated the averages for all the measured physical traits and obtained the *r* values for the relationships between each distance unit and every known individual from the studied species in the plot. This analysis was performed using the R software (R Development Core Team, 2015).

## 3 RESULTS

### 3.1 Leaf defenses: Clustered versus non‐clustered species

**Figure 2:**
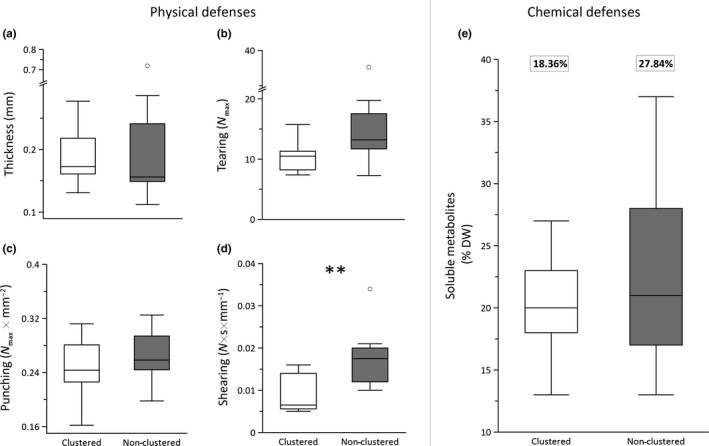
Boxplots showing leaf‐trait comparisons between clustered and non‐clustered groups of species. (a) thickness, (b) tearing, (c) punching and (d) shearing resistance, and (e) percent of mass investment in chemical defenses, DW = dry weight. Horizontal lines in the boxes represent the median of each dataset. Error bars are upper and lower deciles. Results from Mann–Whitney tests showed differences in shearing resistance (*p* < 0.05), but no higher investment in soluble metabolites between clustered and non‐clustered species. Coefficient of variation values (CV%) is shown by a rectangle over columns of soluble metabolites. Significant CV% are in bold (*p* < 0.05; asymptotic test for CV comparisons). Details of Mann–Whitney test results and CV asymptotic test results are shown in Supporting Information Table S2 in Appendix S4

Clustered and non‐clustered species did not differ in thickness, tearing and punching resistance (Figure 2a–c), and mass investment of soluble metabolites (Figure 2e; *p* > 0.05 in all cases; Mann–Whitney test results for median comparisons in Supporting Information Table S2 in Appendix S4). Only shearing test showed significant differences, where clustered species were less resistant to shear than non‐clustered species (Figure 2d; *p* = 0.001). Moreover, clustered species did not show higher variability in chemical defenses than non‐clustered species (further details of CV asymptotic test in Supporting Information Table S2 in Appendix S4).

### 3.2 Physical leaf resistance to damage: saplings versus adult trees

**Figure 3:**
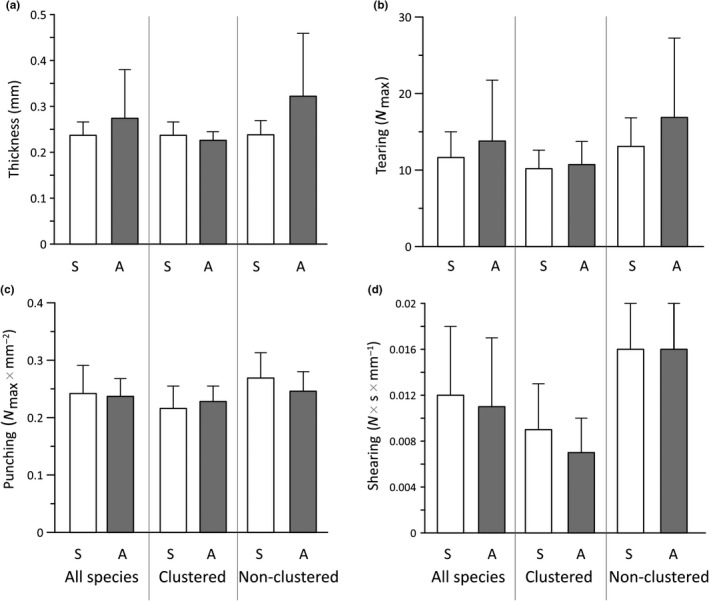
Physical leaf‐trait comparisons between saplings and adult trees for: all species (left columns), clustered species (central columns), and non‐clustered species (right columns). Error bars represent standard deviations. Open boxes represent saplings (S). Solid boxes represent adults (A). *t* Test comparisons showed no differences in leaf resistance between saplings and adults (*p* > 0.05 for all cases). Details of *t* test results are shown in Supporting Information Table S2 in Appendix S5

Physical defenses were similar between saplings and adults for clustered and non‐clustered species and for the pool of all species together (Figure 3; *p* > 0.05 for all cases; further details of *t* test results in Supporting Information Table S2 in Appendix S5). In addition, at the intraspecific level, differences between saplings and adults were only found for *Matisia oblongifolia *(a clustered species) and *Pourouma bicolor* (a non‐clustered species; Supporting Information Table S1 in Appendix S7). *Matisia oblongifolia* showed significant differences in leaf punching resistance, with adult trees showing larger resistance than saplings (on average 0.162 N_max_ × mm^−2^ vs. 0.246 N_max_ × mm^−2^; *p* = 0.005). By contrast, *P. bicolor *showed significant differences in adults versus saplings in: leaf thickness (0.719 mm vs. 0.286 mm; *p* < 0.001), tearing (37.62 N_max_ vs. 17.54 N_max_; *p* < 0.001), and shearing (0.034 N × s × mm^−1 ^vs. 0.019 N × s × mm^−1^; *p* = 0.005).

### 3.3 Quantitative investment in chemical defenses for adults and saplings

**Figure 4:**
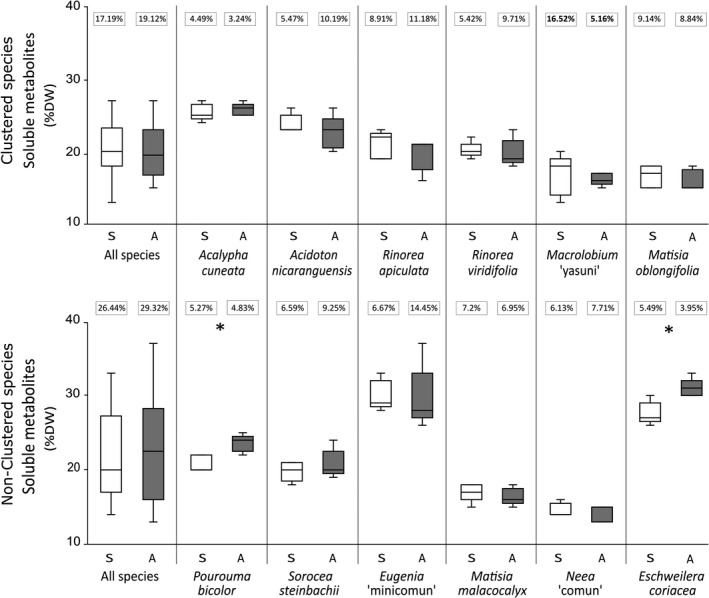
Mass investment in chemical defenses involving soluble metabolites of clustered species and non‐clustered species. Open boxes represent saplings (S). Solid boxes represent adults (A). Horizontal lines in the boxes represent the median of each dataset. Error bars are upper and lower deciles. In y‐axis, DW = dry weight. * denotes statistical significant differences for *p* < 0.01 (*t* tests for intraspecific comparisons). Coefficient of variation values (CV%) are shown by a rectangle over each column. Significant values CV% are in bold (*p* < 0.05; asymptotic test for CV comparisons). Details of *t* tests and asymptotic tests results are shown in Supporting Information Table S1 in Appendix S6

Gravimetric tests showed no significant differences between adults and saplings in chemical defense investment for all clustered and most non‐clustered species (Figure 4). Only *P. bicolor* and *Eschweilera coriacea* (a non‐clustered species) showed an ontogenetic difference in chemical defense investment, with adult trees investing more in chemical defensive compounds than saplings (Figure 4; *p = *0.004 for both species. *t *Test results details in Supporting Information Table S2 in Appendix S6). Only saplings of *Macrolobium* “yasuni” (a clustered species) showed 11.36% more variation in soluble metabolites than their adult counterparts (Figure 4; further details of CV asymptotic test in Supporting Information Table S2 in Appendix S6).

### 3.4 Specificity of physicochemical leaf traits

**Figure 5:**
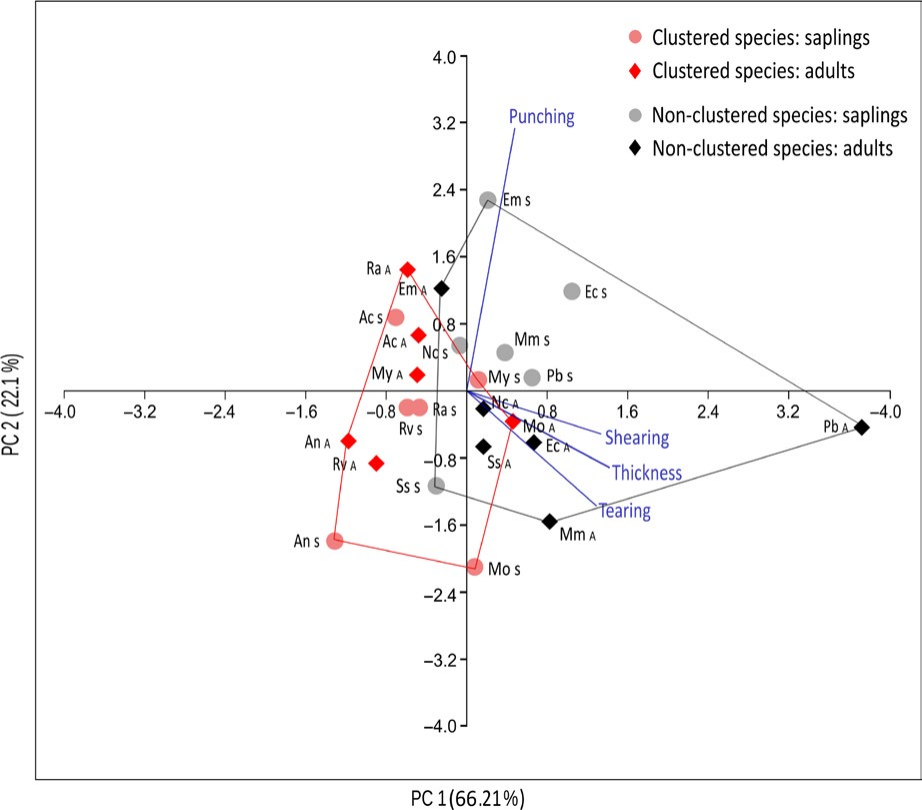
Principal component analysis of the relationship between four physical leaf traits of our 12 focal common tree species: thickness, tearing, punching, and shearing resistance. Ontogenetic stages are differentiated with filled dots (saplings) and diamond symbols (adult trees). Red polygon includes all clustered species. Gray polygon includes all non‐clustered species. Species codes are detailed in Table 1

The PCA model explained 66.21% of the total variance on the first axis, separating the pool of clustered species from the pool of non‐clustered species (Figure 5), where shearing and punching tests were highly correlated. Moreover, the PCA also showed that adults of *P. bicolor* were positively related to higher values of leaf resistance of shearing, thickness, and tearing while higher values of punching resistance where positively related for saplings of *Eugenia* “minicomun” (Figure 5). A detailed inspection of how physical leaf traits vary throughout both ontogenetic stages, a heatmap comparison of leaf resistance values, showed that only adults of *P*. *bicolor *exhibited higher values of leaf thickness, tearing, and shearing compared to saplings (Supporting Information Table S1 in Appendix S8).

**Figure 6:**
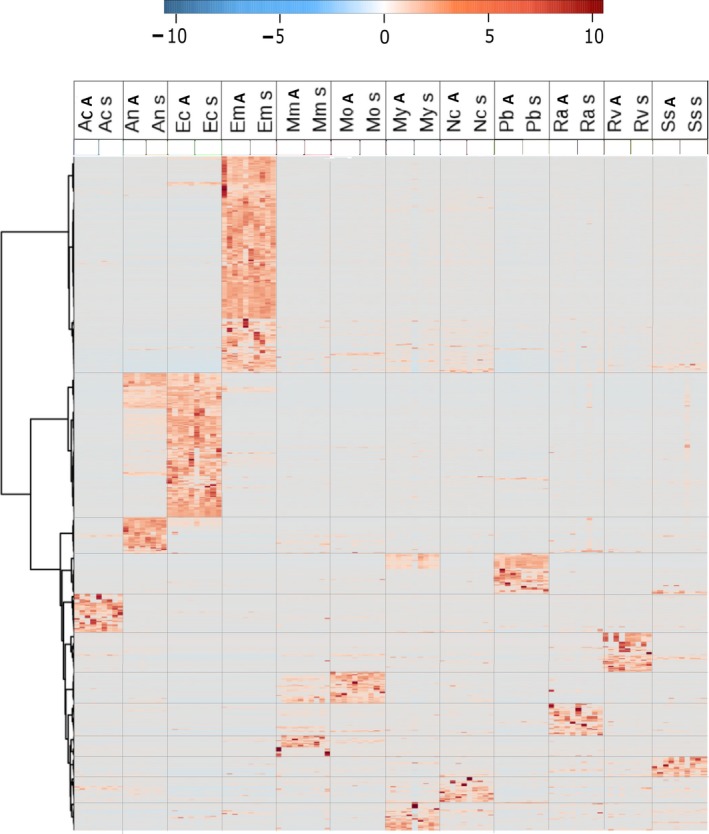
Heatmap of a hierarchical clustering based on relative abundance of soluble metabolites comparing the metabolomic profiles between adults and saplings of our 12 focal common tree species. Each column represents a treatment per species (sapling and adult). Each row represents a metabolite with a unique *m/z* (mass to charge ratio) and retention time. Dendrogram along the left side shows chemistry similarities on metabolites. The color scale for metabolite relative abundance is based on signal intensity (total ion current from the mass spectrometer) where dark red means high abundance of a given metabolite, white means intermediate abundance, and blue means low abundance. Saplings and adults are denoted by “S” and “a.” Species codes are detailed in Table 1

Quantitative analyses of the UPLC‐MS metabolomics data for clustering showed significant differences in secondary metabolite identity between species (Figure 6 and Supporting Information plates S1a‐l in Appendix S11) but not between ontogenetic stages. Similarly, PCA analyses for the pool of species did not show any grouping patterns according to the ontogenetic stage, showing similar chemical profiles within species (Supporting Information Figure S1 in Appendix S9; Figure S1 in Appendix S10, and Appendix S11).

### 3.5 Relationships between spatial clustering and physicochemical leaf‐trait variability

Increasing variation in thickness and punching resistance for adults was positively correlated with spatial clustering (Supporting Information Figure S1a,c in Appendix S12). That is, adults of most densely distributed species show higher resistance and greater variation than their saplings for these two physical traits. The remaining variables showed no significant relationships with clustering levels (Supporting Information Figure S1b,d in Appendix S12).

### 3.6 Relationships between physical leaf traits and spatial clustering at local and study‐plot scales

Most of the physical traits were not related to spatial clustering, either for saplings or adult trees (Supporting Information Figure S1a–c in Appendix S13; linear and non‐linear regressions showed *R* ^2^ < 0.215 and *p* > 0.05 in all cases). However, shearing leaf resistance of saplings and adult trees was negatively related to spatial clustering (Supporting Information Figure S1d in Appendix S13; saplings: *R* ^2^ = −0.375, *p = *0.034; adults: *R* ^2^ = −0.457, *p = *0.016), with most densely distributed species showing less investment of leaf shearing resistance, contrary to our expectations.

**Figure 7:**
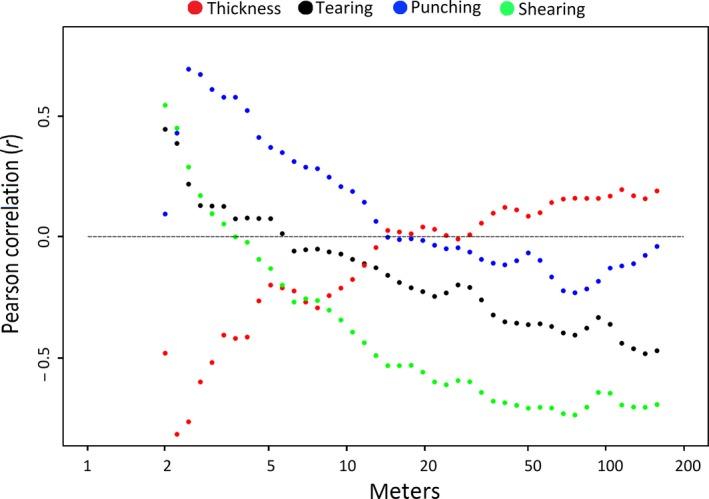
Scatter plot showing the Pearson correlation values (*Y*‐axis) between physical leaf traits and the Euclidean distance of surrounding conspecific neighbors at the level of the 50‐ha. studied plot. Dots above the gray dashed line are positive correlations while dots below are negative correlations. Physical leaf traits are distinguished by colors, where red is leaf thickness, black is tearing resistance, blue is punching resistance, and green is shearing resistance

Regardless the cluster status of our 12 focal species, at the plot scale, we found leaf resistance to shear and punch was larger (Pearson *r* > 0.5) at distances ≤2 m and ≤5 m, respectively. Tearing resistance was larger at ~2.5 m. In contrast, leaves showed to be thicker from ≥2.5 m of conspecific neighboring (Figure 7).

## 4 DISCUSSION

### 4.1 Leaf physicochemical defenses are not different ontogenetically between clustered and non‐clustered species

Contrary to our three hypotheses, physicochemical defenses were similar between clustered and non‐clustered species and for both ontogenetic stages, except for *P. bicolor*, where saplings showed lower resistance than adults. Remarkably, chemical defenses were, however, clearly different at interspecific level. Considering that plants may be fully defended in the adulthood (Boege & Marquis, 2005), our results may suggest that (a) plants in this forest reach their maximum defensive levels since the sapling stage, and (b) chemical defensive traits are extremely species specific potentially to deter specific pathogens (Dyer et al., 2007; but cf. Carmona et al., 2011; Novotny et al., 2010).

The defensive and competitive strategy of any species may depend on the particular herbivore pressure each species is submitted to, and the microenvironment where it grows (Fine et al., 2006; Janzen, 1970). Following Cárdenas et al. (2014), we agree that plant functional traits, such as leaf defenses, may be a complex mosaic of action/reaction between plants (conspecific or not) and natural enemies, where trees in the Yasuní might fall along tolerance/escape/defense strategies due to intrinsic physiological constraints (Cárdenas et al., 2014; Kursar & Coley, 2003). Within this context, we offer two potential explanations how plant species of this plant community could balance the cost–benefit relationship between plant defense investments and resource allocation.

First, saplings of non‐clustered species are most likely to be associated and deeply adapted to seed dispersal over large distances to escape from density‐dependent negative effects (Augspurger, 1984; Bagchi et al., 2011; Lopes, 2007; Valencia, Foster, et al., 2004; Wright, 2002). Consequently, seeds sprout far from their conspecific adults and offspring and would not be directly attacked by natural enemies, favoring the recruitment and establishment of individuals of these species (Metz et al., 2010). Competition for resources may be nearly null among relatively distant conspecifics (Comita et al.., 2014; Johnson et al., 2012; Peters, 2003; Wright, 2002). However, this does not explain why some non‐clustered species such as *Matisia malacocalyx*, *Neea* “comun,” and *P. bicolor* present significative large values of leaf damage (see Cárdenas et al., 2014). Are the survival strategies of these species more related to damage tolerance and a fast growing strategy (Boege & Marquis, 2005; Boyden, Reich, Puettmann, & Baker, 2009)? Plants could allocate resources to growth as a priority response against herbivore pressure for rapid tree establishment and plant higher fitness (Boege et al., 2011; Boege & Dirzo, 2004). Our estimations of the relative growth rate (RGR) for each tree species (Supporting Information Table S1 in Appendix S14) show that, on average, the RGR of saplings of non‐clustered species was slightly larger than clustered species, although these results were not significant (non‐clustered species = 0.034 mm × year^−1^ vs. clustered species = 0.022 mm × year^−1^; *p* = 0.097; *t* = −1.831; results not shown). Furthermore, only the RGR of *P. bicolor* saplings (0.054 mm × year^−1^) is high above the RGR sapling average (0.028 ± 0.011 mm × year^−1^). Nevertheless, these results suggest that non‐clustered species may potentially compensate the foliar tissue loss caused by herbivores via internal mechanisms that involve modifications of plant metabolism, as well as, external mechanisms of the plant environment that are favorable to plant growth and yield (McNaughton, 1983; but cf. Fornoni, 2011). Such a response may depend on whether plants have induced or constitutive defenses and/or are attacked by generalists or specialist enemies (Fornoni, 2011; Huang et al., 2010).

Second, clustered species may show a suite of ecological and evolutionary traits that allow them to defy the CNDD. Poor seed dispersal favors their clustering coexistence (Peh et al., 2011), a particular characteristic shared by our focal clustered species (Bagchi et al., 2011; Silva & Souza‐Lima, 2013). Seeds of clustered species are adapted to sprout in the understory near to conspecific adults and reach adulthood in the same microhabitat (Corrales et al., 2016; Peh et al., 2011; Torti, Coley, & Kursar, 2001). Mechanisms for such adaptation as we hypothesized in our study may be that saplings of clustered species have developed better defenses when compared to conspecific adults and saplings of non‐clustered species. Lack of significant differences in defense traits at the early ontogenetic level of our clustered species may suggest that nutrient allocation in defenses may not be the principal strategy to survive in aggregated conditions. Other mechanisms such as mycorrhizal symbiosis may guarantee nutrient availability that is supplied at both ontogenetic stages, potentially through higher rates of litter decomposition in synergy with specialized biota in their surroundings (Corrales et al., 2016; Gholz, Wedin, Smitherman, Harmon, & Partons, 2000; Peh et al., 2011; Torti et al., 2001).

### 4.2 Variation in secondary metabolite investment at intra‐ and interspecific level

At intraspecific level, only adults of *P. bicolor *and *E. coriacea* (two non‐clustered species) invested threefold in soluble metabolites when compared to saplings. These two species may follow the prediction of Boege and Marquis (2005) that mature plants in non‐reproductive stages would reach the maximum defense and tolerance rates, while resource allocation at juvenile stages may be for growth and leaf production instead of defenses against pathogens. However, there were 10 other species that did not follow Boege and Marquis (2005) hypothesis. According to the literature, due to physiological constraints, plants have a variety of life‐history defense strategies that originated from a particular evolutionary trajectory, resulting in a convergent evolution of defenses (Kursar & Coley, 2003). Thus, a plant will guarantee its survival by combinations of traits, such as physiological, chemical, and phenological traits (e.g., synchronous leaf production, rapid leaf expansion, and high leaf turnover rates), which have also been shown to be negatively correlated with herbivory (Cárdenas et al., 2014; Kursar & Coley, 2003). This suggests that trees may fall along a tolerance/escape/defense continuum strategy to survive and become established in the forest. Further tests of plant defense trade‐offs (e.g., growth rate vs. herbivore susceptibility; inductive/constitutive defenses vs. generalist/specialist herbivores) and young leaf color pattern (Queenborough et al., 2013) should be studied in the future for a better understanding of defense mechanisms in plants.

The diversity (i.e., variability) of chemical compounds has shown to be effective against natural enemies (Agrawal & Weber, 2015; Richards et al., 2015), and the greater diversity of secondary metabolites in young leaves has been associated with low herbivory rates (Kursar & Coley, 2003). Because *E*.* “*minicomun,” *E. coriacea*, *Acidoton nicaraguensis, *and *Acalypha cuneata* showed chemical dissimilarities and the largest number of metabolites compared to the remaining species, it is likely they would be less affected by natural enemies. In fact, Cárdenas et al. (2014) found that *E. “*minicomun” presented 2.84% on average of leaf damage (among the lowest rates of herbivory in that study), caused by herbivores because of small leaf size and high investment in physical defenses such as punching resistance. Accordingly, our results show that *E.* “minicomun” would also escape from herbivores by their more robust chemical properties of essential oils, which have antibacterial and antifungal effects, a common characteristic of Myrtaceae family (Nascimento, Locatelli, Freitas, & Silva, 2000; Sohilait, 2015). If we consider, however, that the mean herbivore damage in the 28 studied species was of 13.4% ± 5.9 (Cárdenas et al., 2014) and that *E. coriacea* showed 8.02% on average and *A. nicaraguensis* showed 13.34%, one may agree that, at the community level, plant traits other than secondary metabolites may also strongly predict herbivore susceptibility in tropical hyperdiverse forests (Carmona et al., 2011; and c.f. López‐Goldar et al., 2018). Analyses at the individual level will reveal the specific role of physicochemical plant traits on deterring pathogens at particular circumstances directly and indirectly related to the temporal and spatial variable organisms are growing (environmental seasonality, soil nutrients, neighborhood, herbivores outbreak intensity, microhabitat, among others).

### 4.3 Distance between conspecifics is correlated with physical leaf traits

Metz et al. (2010) found a strong evidence of the CNDD effect in the same study area, where an increasing abundance of conspecific trees in the neighborhood of seedlings led a strong negative impact on first‐year plant survival. They suggest that neighborhood diversity favors survival and establishment of species with low phylogenetic relatedness because they represent “null” competition to recently established seedlings and do not attract specialized pathogens. Unexpectedly, regardless of whether plants were categorized as clustered or not, we found that plants tend to have thinner leaves and high levels of physical leaf defenses at small distances between conspecific neighbors. Conversely, plants tend to have thicker leaves at greater distances between conspecifics. This suggests that more proximal conspecifics would be better defended, while conspecifics far apart from each other could invest more in growth or reproduction. Interactions between conspecifics may favor the investment of certain functional traits related to physical defenses (Kunstler et al., 2015), counteracting the effects of the CNDD.

### 4.4 Leaf thickness: a physical barrier or part of nutrient content?

From a biological standpoint, leaf thickness is correlated with leaf toughness, photosynthetic capacity (Schulze, Kelliher, Korner, Lloyd, & Leuning, 1994), and nitrogen content (Kitajima & Poorter, 2010; Niinemets, 1999; Witkowski & Lamont, 1991), which, in turn, is related to leaf nutritional quality (Coley & Barone, 1996; Herms & Mattson, 1992). In spite that literature has constantly shown that thinner leaves would be more palatable to herbivores (Onoda et al., 2011; Wright & Cannon, 2001), our study demonstrate that closer, conspecific trees have thinner leaves than more distant trees. This may suggest that thinner leaves may be a defense strategy to avoid herbivores coming from the closer, conspecific, adult hosts by investing less in nutritious leaves (i.e., less fleshy). In contrast, thicker leaves of conspecifics that are far apart from each other may be richer in nutrients (i.e., more fleshy) because they have less probability to be attacked by natural enemies. We suggest that leaf thickness should be better considered as a template of leaf nutritional quality rather than a functional trait of resistance agreeing with Kitajima and Poorter (2010) and Witkowski and Lamont (1991).

## 5 CONCLUSIONS

Except for few particular cases, physicochemical leaf traits related to antiherbivore defenses did not support any of our hypotheses. Our results suggest that leaf antiherbivore defenses and leaf defense trade‐offs may not be a pivotal strategy for explaining survival of clustered species. In contrast, trees in Yasuní may fall along a gradient of tolerance/escape/defense strategies for each species, physiological constraint to survive and become established in the forest. We conclude that other mechanisms, such as those related to soil nutrient exploitation efficiency (through specialized detritivores or decomposers, and symbiotic associations such as mycorrhizae), volatile organic compounds, and herbivory tolerance may be more “competitively advantageous” to counteract the effects of the CNDD for both clustered and non‐clustered species. Those mechanisms shall be evaluated to understand conspecific coexistence in this hyperdiverse forest.

## CONFLICT OF INTEREST

None declared.

## AUTHOR CONTRIBUTIONS

JC‐Q made the field work, collected, and analyzed data, wrote and edited the manuscript. MJE supported chemical analyses and made suggestions for data analyses and manuscript edition. RV provided necessary information of the YFDP, made suggestions for data analyses and manuscript edition. DM supported the field work. REC conceived the ideas, designed the methodology, made the field work, helped with data analyses, wrote, and edited the manuscript.

## Data Availability

Additional Data Accessibility may be found in the online version of this article.
