# Supplementary material for: Physical, but not chemical, antiherbivore defense expression is related to the clustered spatial distribution of tropical trees in an Amazonian forest
Source: Ecol Evol. 2019 Jan 31;9(4):1750–63. doi: 10.1002/ece3.4859 (PMC6392389; doi:10.1002/ece3.4859)
Supplement: Supplementary file 1 [file ECE3-9-1750-s001.docx]

# **APPENDIX S1 - Yasuní Forest Dynamic Plot (YFDP)**


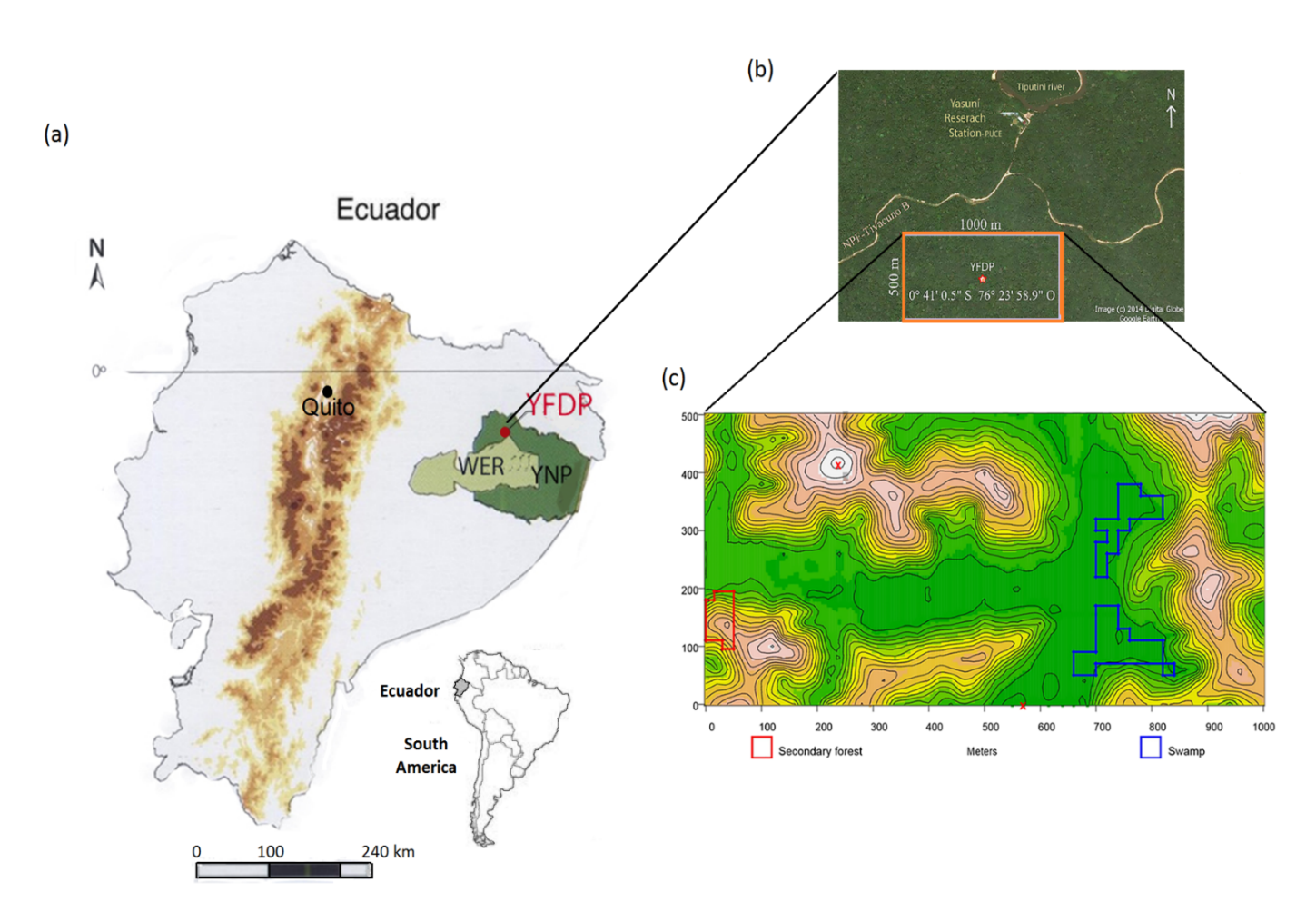


### **Figure S1** Location of the study area in the western eacuadorian amazon. (a) Map of continental Ecuador. Dark green area represents the Yasuní National Park. Light green area represents the Waroni Ethnic Territoy (WER). Red dot represents "Yasuní Forest Dynamics Plot (YFDP)". (b) Location of the 50 ha "Yasuní Forest Dynamics Plot (YFDP)" near to the Yasuní Research Station. Area plot is represented by an orange rectangle. (c) Topographic map of the 50 ha Project "Yasuní Forest Dynamics Plot (YFDP)" (1000 х 500 m), ranging 215–248 m above sea level. The distance between the contour lines is 2 m in altitude. Red polygon represents a part of secondary forest, and the blue polygons represent two swamps present in the plot. The green shades are for valleys. Brown and yellowish brown shades are for hills. Pink shades are for higher ridge points. The highest point (248m) and the lowest point (215m) are indicated by red X symbols. Image modified from Pérez *et al*. (2014) and Valencia *et al*. (2004a).

### **References**

Pérez. A.J.. Hernández. C.. Romero-Saltos. H. & Valencia. R. (2014) Árboles emblemáticos de Yasuní Ecuador 337 especies. Publishing by QCA Herbarium. School of Biological Science. Pontificia Universidad Católica del Ecuador. Quito.

Valencia. R.. Foster. R.B.. Villa. G.. Condit. R.G.. Svenning. J.C.. Hernandez. C.. Romoleroux. K.. Losos. E.C.. Magard. E. & Balslev. H. (2004a) Tree species distributions and local habitat variation in the Amazon: large forest plot in eastern Ecuador. *Journal of Ecology*, 92, 214–229.

# **APPENDIX S2 – Averages of the diameter at breast height (dbh) of the 12 common species**

**Table S1.** The table shows the minimum and maximum dbh values, the dbh average () for each tree species, and the 95^th^ percentile based on the YFDP data from plot census. 95^th^ percentile was used to define whether an individual should be considered as sapling or adult. Dbh data were recorded in mm. Overlapping values in the dbh max and min columns correspond to trees that reach the minimum dbh of an adult tree, but they are still considered as saplings because they did not reach the reproductive stage yet. Analyses were performed using R software (R Development Core Team, 2017).

| **Species name** | **dbh 5^th^ – 95^th^ percentiles** | | **dbh**  **min** | **dbh**  **max** | **** | **Stage** |
| --- | --- | --- | --- | --- | --- | --- |
| *Acalypha cuneata* | | 18.52 | 8.57 | 19.75 | 13.91 | sapling |
|  |  | 36.68 | 19.30 | 39.17 | 27.68 | adult |
| *Acidoton nicaraguensis* | | 12.67 | 4.46 | 12.67 | 8.49 | sapling |
|  |  | 29.87 | 17.83 | 31.67 | 22.80 | adult |
| *Rinorea viridifolia* | | 29.92 | 9.70 | 32.42 | 19.18 | sapling |
|  |  | 148.50 | 48.11 | 151.00 | 76.84 | adult |
| *Rinorea apiculata* | | 25.75 | 9.81 | 27.15 | 18.55 | sapling |
|  |  | 104.99 | 46.00 | 131.50 | 72.37 | adult |
| *Macrolobium* 'yasuni' | | 33.567 | 8.68 | 33.72 | 21.29 | sapling |
|  |  | 119.83 | 49.00 | 125.00 | 77.77 | adult |
| *Matisia oblongifolia* | | 20.67 | 10.77 | 21.11 | 14.51 | sapling |
|  |  | 55.00 | 23.62 | 58.00 | 40.65 | adult |
| *Pourouma bicolor* | | 106.95 | 24.36 | 129.00 | 56.27 | sapling |
|  |  | 435.25 | 174.00 | 478.00 | 288.10 | adult |
| *Sorocea steinbachii* | | 38.54 | 7.24 | 40.38 | 25.24 | sapling |
|  |  | 192.00 | 30.98 | 240.00 | 84.03 | adult |
| *Eugenia* 'minicomun' | | 34.98 | 10.72 | 37.17 | 19.88 | sapling |
|  |  | 187.75 | 28.96 | 226.00 | 93.36 | adult |
| *Matisia malacocalyx* | | 68.10 | 19.39 | 69.00 | 40.83 | sapling |
|  |  | 162.08 | 79.08 | 196.50 | 111.10 | adult |
| *Neea* 'comun' | | 50.62 | 14.50 | 57.00 | 28.54 | sapling |
|  |  | 203.59 | 63.00 | 217.00 | 133.30 | adult |
| *Eschweilera coriacea* | | 60.28 | 16.22 | 69.91 | 36.70 | sapling |
|  |  | 362.25 | 120.00 | 405.00 | 212.80 | adult |

# **APPENDIX S3 – Metabolomic profile analyses: ten vs. five samples per treatment for each tree species**

Preliminary chromatographic analyses were performed to detect the optimal number of samples required to analyse the similarities or differences in chemical compounds involving secondary soluble metabolites of leaves between saplings and adult trees. For that, we just randomly chose two species: *Rinorea apiculata* and *Acidoton nicaraguensis* and analysed 10 samples (20 samples in total) and then five samples (10 samples in total) for each of the two ontogenetic stages.

Soluble metabolites were analysed using ultra-performance liquid chromatography and mass spectrometry (UPLC-MS) with an Acquity UPLC® I-Class system and a Xevo® G2 Q-ToF MS equipped with LockSpayTM (Waters. Milford. MA). For Liquid Chromatographic conditions, 2 µL of sample were injected on a Acquity® BEH C18 column (2.1 x 150 mm) fitted with VanGuard Precolumn (2.1 x 5 mm; Waters. Milford. MA). The column temperature was kept constant at 40 °C, and the solvents were MS grade (Optima® LC/MS grade, Fisher Scientific, Waltham. MA). Solvent A was water + 0.1% formic acid and solvent B was acetonitrile + 0.1% formic acid. The flow rate was 0.5 mL/min. Mass spectra were acquired in negative ionization and sensitivity modes using the following parameters: m/z (mass to charge ratio) of 50–5000 Da, centroid mode, capillary voltage 2.40 Kv, sampling cone voltage 30 V, extraction cone voltage 4 V, source temperature 100 °C, desolvation gas temperature 400 °C, desolvation gas flow 600 L/h.

Dendrogram trees were required to assess the significance of analyses. *P*-values of the uncertainty assessment are generated by calculating approximately unbiased (AU) and the bootstrap probability (PB), which is the frequency that appears in bootstrap replicates (Suzuki & Shimodaira, 2006).

We used the library ‘muma’ for the principal component analyses, followed by hierarchical cluster analysis, using the library ‘pvclust’ (Wiggins *et al*., 2016). Subsequently, to calculate a statistical measure of confidence for the selected clusters (an unbiased confidence level), hierarchical cluster dendrogram was performed with bootstrap iterations. For model selection, we used a cophonetic correlation, which means, the relationship between the original distance matrix and the binary matrix representing the partitions in the cluster diagram. The clustering algorithm (i.e. single. ward. complete. centroid. and median) with the highest cophonetic correlation was selected as the best one for each comparison.

PCAs and dendrogram results of the two species did not indicate metabolomic differentiation among saplings and adults when we analysed ten or five samples. Thus, for each tree species of the study. we only used five samples per treatment to perform chemical leaf trait analyses. For analyses with five samples. we also randomly chose five samples from the pool of 10. This was repeated by three times with a different set of five samples per treatment.

**a)** **Principal Component Analysis of *Acidoton nicaraguensis***

**
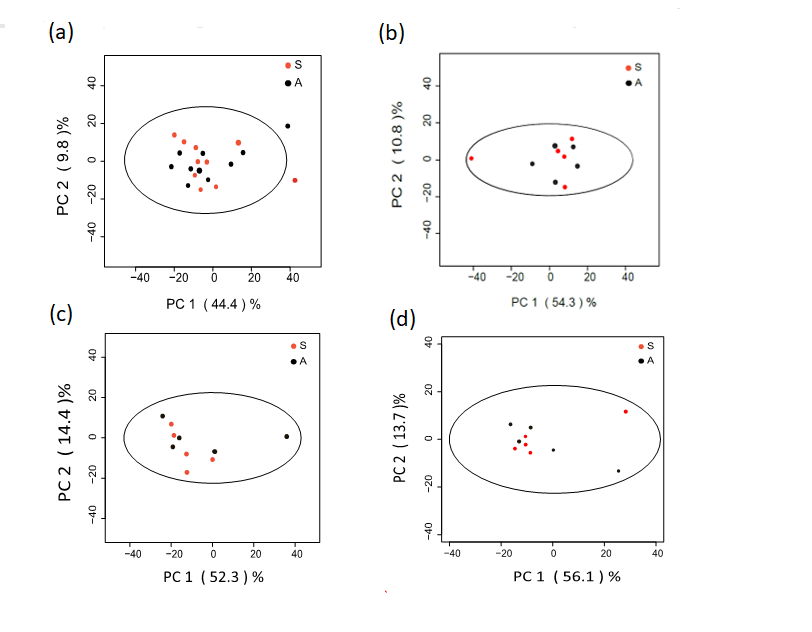
**

### **Fig. S1-a**. (a) PCA of *A. nicaraguensis* containing ten samples per ontogenetic state did not indicate metabolomic differentiation between saplings and adults. (b), (c) and (d) PCAs containing five samples per ontogenetic state did not indicate metabolomic differentiation between saplings and adults. Values are derived from the total ion current of features obtained from UPLC-MS analysis. S = saplings. A = adults.

**b)** **Dendrogram of *Acidoton nicaraguensis***


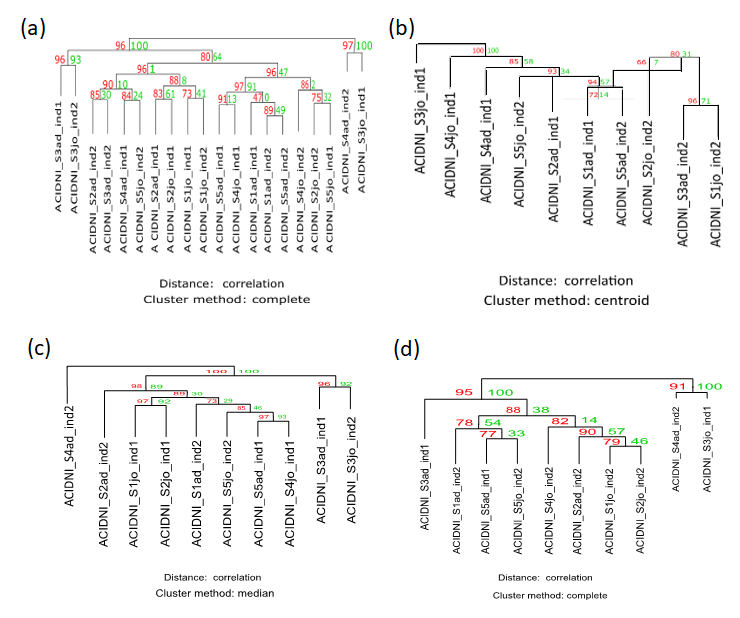


### **Fig. S1-b**. (a) Hierarchical clustering of *A. nicaraguensis* containing ten samples per ontogenetic state did not show any pattern of metabolomic differentiation between adult trees and saplings. (b), (c) and (d) PCAs containing five samples per ontogenetic state did not indicate metabolomic differentiation between saplings and adults. Values on the left side of the branch are the approximately unbiased *P*-values (AU) represented by red colour. Values on the right side of the branch are the bootstrap probability *P*-values (PB) represented by green colour. Clusters with *P*-values of AU ≥ 95 are significant at 0.05

**(c)** **Principal Component Analysis of *Rinorea apiculata***


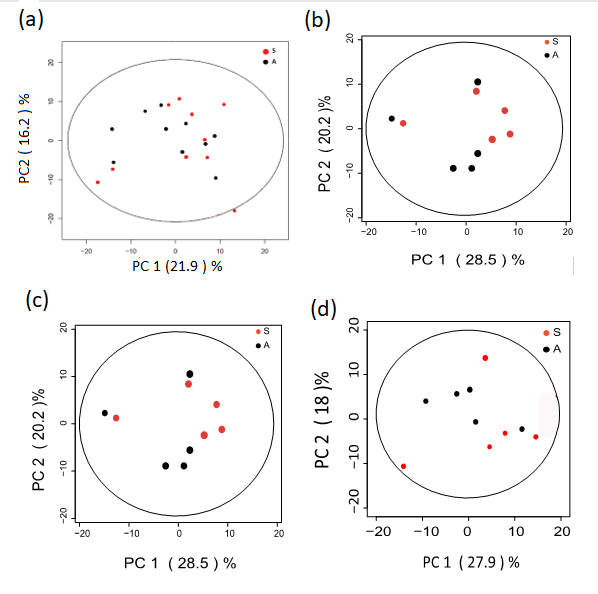


### **Fig. S2-a**. (a) PCA of *R. apiculata* containing ten samples per ontogenetic state did not indicate metabolomic differentiation among saplings and adults. (b), (c) and (d) PCAs containing five samples per ontogenetic state did not indicate metabolomic differentiation among saplings and adults. Values are derived from the total ion current of features obtained from UPLC-MS analysis. S = saplings. A = adults.

**(d)** **Dendrograms of *Rinorea apiculata***

**
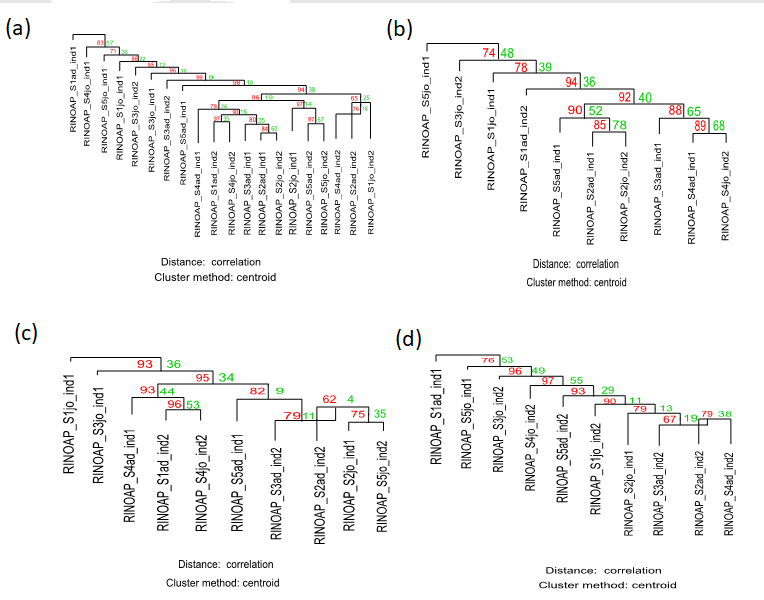
**

### **Fig. S2-b**. (a) Hierarchical clustering of *R. apiculata* containing ten samples per ontogenetic state did not show any pattern of metabolomic differentiation between adult trees and saplings. (b), (c) and (d) PCA. containing five samples per ontogenetic state did not indicate metabolomic differentiation among saplings and adults. Values on the left side of the branch are the approximately unbiased *P*-values (AU) represented by red colour. Values on the right side of the branch are the bootstrap probability *P*-values (PB) represented by green colour. Clusters with *P*-values of AU ≥ 95 are significant at 0.05.

**References**

Wiggins. N. L. Forrister. D. L.. Endara. M. J.. Coley. P. D.. & Kursar. T. A. (2016) Quantitative and qualitative shifts in defensive metabolites define chemical defense investment during leaf development in Inga. a genus of tropical trees. *Ecology and Evolution*, **6** (2), 478–492.

Suzuki.R & Shimodaira. H. 2006. Pvclust: a R package for assessing the uncertainty in hierarchical clustering. *Bioinformatics*. **22** (12). 1540-1542.

# **APPENDIX S4 –** **Statistical analyses for physico-chemical traits comparisons between clustered and non-clustered species**

### **Table S1.** **Normality test for physico-chemical traits comparisons between clustered and non-clustered species.** Prior to evaluate the differences in leaf resistance and leaf chemistry investment between clustered and non-clustered species (without including their ontogenetic stages), we performed Shapiro-Wilk normality tests to evaluate the type of data distribution. The Table below shows Shapiro-Wilk results to confirm normal distributions of the residuals. *w*= W-value, *P= p*-value (where *P* ≤ 0.05 means significant difference to normal distribution ), and N = number of sampled individuals. Analyses were performed in PAST software v3.07 (Hammer *et al*., 2001).

| **Traits** | | **Type of clustering status** | **N** | ***w*** | ***P*** |
| --- | --- | --- | --- | --- | --- |
| Physical | Thickness (mm) | Clustered species | 116 | 0.571 | **1.93E-23** |
|  |  | Non-clustered species | 120 | 0.609 | **7.46E-22** |
|  | Tearing (N_max_) | Clustered species | 120 | 0.919 | **4.64E-10** |
|  |  | Non-clustered species | 120 | 0.832 | **1.90E-14** |
|  | Punching (N_max_ × mm^-2^) | Clustered species | 120 | 0.934 | **1.18E-08** |
|  |  | Non-clustered species | 120 | 0.944 | **2.46E-07** |
|  | Shearing (N × s × mm^-1^) | Clustered species | 120 | 0.894 | **1.07E-11** |
|  |  | Non-clustered species | 120 | 0.899 | **7.31E-11** |
| Chemical | Mass investment in soluble metabolites (%DW) | Clustered species | 52 | 0.985 | **0.039** |
|  |  | Non-clustered species | 52 | 0.946 | **0.011** |

**Table S2.** **Mann Whitney test results for physico-chemical traits comparisons between clustered and non-clustered species.**

Based on Shapiro-Wilk results, we performed Mann Whitney tests for median comparisons to assess the significance of physico-chemical leaf traits comparisons between clustered and non-clustered species. For physical traits, N = average of a given measured trait for each studied species per stage. For chemical trait, N = number of used samples to analyse soluble metabolites. We also assessed the equality of coefficient of variation (CV) of the population using the asymptotic test. Mann Whitney analyses were performed in PAST statistical software v3.07 (Hammer *et al*., 2001). Asymptotic tests (Feltz & Miller, 1996; Krishnamoorthy & Lee, 2014) were performed in R software using the library “cvequality” (R Development Core Team, 2017). P_AD = *p*-value of the asymptotic test.

| **Traits** | | **Type of clustering status** | **N** | **** | **U** | ***P*** | **CV** | **AD** | ***P*_AD** |
| --- | --- | --- | --- | --- | --- | --- | --- | --- | --- |
| Physical | Thickness | Clustered species | 12 | 0.234 | 61 | 0.237 |  |  |  |
|  | (mm) | Non-clustered species | 12 | 0.277 |  |  |  |  |  |
|  | Tearing | Clustered species | 12 | 12.35 | 38 | 0.908 |  |  |  |
|  | (N_max_) | Non-clustered species | 12 | 13.79 |  |  |  |  |  |
|  | Punching | Clustered species | 12 | 0.242 | 56 | 0.954 |  |  |  |
|  | (N_max_ × mm^-2^) | Non-clustered species | 12 | 0.246 |  |  |  |  |  |
|  | Shearing | Clustered species | 12 | 0.012 | 16.5 | **0.001** |  |  |  |
|  | (N × s × mm^-1^) | Non-clustered species | 12 | 0.013 |  |  |  |  |  |
| Chemical | Mass investment in soluble metabolites (%DW) | Clustered species | 52 | 19.52 | 1553.5 | 0.154 | 18.36 | 9.241 | **0.002** |
|  |  | Non-clustered species | 52 | 22.08 |  |  | 27.84 |  |  |

**References**

Feltz. C. J.. & Miller. G. E. (1996) An asymptotic test for the equality of coefficients of variation from k populations. *Statistics in Medicine*. **15** (6). 647-658. [https:// 10.1002/(SICI)1097-0258(19960330)15:6<647::AID-SIM184>3.0.CO;2-P](https://w)

Hammer. Ř.. Harper. D.A.T.. Ryan. P.D. 2001. PAST: Paleontological statistics software package for education and data analysis. *Palaeontologia Electronica* 4(1): 9pp. <http://palaeo-electronica.org/2001_1/past/issue1_01.htm>.

Krishnamoorthy. K.. & Lee. M. (2014) Improved tests for the equality of normal coefficients of variation. *Computational Statistics*. **29** (1-2). 215-232. <http://10.1007/s00180-013-0445-2>

# **APPENDIX S5 –** **Statistical analyses for physical traits of saplings and adult trees of the 12 common species**

### **Table S1.** **Normality tests for physical for physical traits of saplings and adult trees** Prior to *t*-test comparisons between saplings and adults. We made sure residuals from our data showed normal distributions according to both ontogenetic stages using Shapiro-Wilk normality test (*w*-value). *P*-values > 0.05 confirm normal distributions of the residuals. All analyses were performed in PAST statistical software v3.07 (Hammer *et al*., 2001).

| **Physical trait** | **Comparisons** | **Stages** | **N** | ***w*** | ***P*** |
| --- | --- | --- | --- | --- | --- |
| Thickness  (mm) | All species | saplings | 120 | 0.969 | 0.907 |
|  |  | adults | 120 | 0.589 | 0.080 |
|  | Clustered species | saplings | 60 | 0.934 | 0.618 |
|  |  | adults | 60 | 0.900 | 0.374 |
|  | Non-clustered species | saplings | 60 | 0.898 | 0.364 |
|  |  | adults | 60 | 0.657 | 0.060 |
| Tearing  (N_max_) | All species | saplings | 120 | 0.929 | 0.375 |
|  |  | adults | 120 | 0.739 | 0.054 |
|  | Clustered species | saplings | 60 | 0.913 | 0.458 |
|  |  | adults | 60 | 0.889 | 0.315 |
|  | Non-clustered species | saplings | 60 | 0.969 | 0.886 |
|  |  | adults | 60 | 0.837 | 0.124 |
| Punching  (N_max_ × mm^-2^) | All species | saplings | 120 | 0.961 | 0.801 |
|  |  | adults | 120 | 0.917 | 0.267 |
|  | Clustered species | saplings | 60 | 0.864 | 0.204 |
|  |  | adults | 60 | 0.926 | 0.556 |
|  | Non-clustered species | saplings | 60 | 0.958 | 0.805 |
|  |  | adults | 60 | 0.936 | 0.630 |
| Shearing  (N × s × mm^-1^) | All species | saplings | 120 | 0.913 | 0.234 |
|  |  | adults | 120 | 0.906 | 0.190 |
|  | Clustered species | saplings | 60 | 0.749 | **0.019** |
|  |  | adults | 60 | 0.776 | 0.095 |
|  | Non-clustered species | saplings | 60 | 0.875 | 0.246 |
|  |  | adults | 60 | 0.876 | 0.248 |

### **Table S2.** **Physical leaf trait comparisons between saplings and adult trees**

### *t*- test table for physical leaf trait comparisons between saplings and adults within clustered. non-clustered species and all species (clustered + non-clustered species). *t*-test analyses were performed in PAST statistical software v3.07 (Hammer *et al*., 2001).

| **Physical Trait** | **Clustering type** | **Stage** | **N** | **** | ***t*** | ***P*** |
| --- | --- | --- | --- | --- | --- | --- |
| Thickness (mm) | All species | Saplings | 120 | 0.234 | -1.377 | 0.182 |
|  |  | Adults | 120 | 0.277 |  |  |
|  | Clustered | Saplings | 60 | 0.229 | -0.182 | 0.859 |
|  |  | Adults | 60 | 0.231 |  |  |
|  | Non-clustered | Saplings | 60 | 0.238 | -1.459 | 0.078 |
|  |  | Adults | 60 | 0.323 |  |  |
| Tearing  (N_max_) | All species | Saplings | 120 | 12.35 | -0.577 | 0.569 |
|  |  | Adults | 120 | 13.79 |  |  |
|  | Clustered | Saplings | 60 | 10.177 | -0.335 | 0.744 |
|  |  | Adults | 60 | 10.708 |  |  |
|  | Non-clustered | Saplings | 60 | 13.095 | -0.839 | 0.421 |
|  |  | Adults | 60 | 16.872 |  |  |
| Punching (N_max_ × mm^-2^) | All species | Saplings | 120 | 0.242 | -0.227 | 0.822 |
|  |  | Adults | 120 | 0.246 |  |  |
|  | Clustered | Saplings | 60 | 0.215 | -1.234 | 0.245 |
|  |  | Adults | 60 | 0.241 |  |  |
|  | Non-clustered | Saplings | 60 | 0.268 | 0.828 | 0.427 |
|  |  | Adults | 60 | 0.25 |  |  |
| Shearing  (N × s × mm^-1^ | All species | Saplings | 120 | 0.012 | -0.399 | 0.693 |
|  |  | Adults | 120 | 0.013 |  |  |
|  | Clustered | Saplings | 60 | 0.008 | 0.083 | 0.935 |
|  |  | Adults | 60 | 0.008 |  |  |
|  | Non-clustered | Saplings | 60 | 0.017 | -0.615 | 0.541 |
|  |  | Adults | 60 | 0.018 |  |  |

**References**

Hammer. Ř.. Harper. D.A.T.. Ryan. P.D. 2001. PAST: Paleontological statistics software package for education and data analysis. *Palaeontologia Electronica* 4(1): 9pp. <http://palaeo-electronica.org/2001_1/past/issue1_01.htm>.

# **APPENDIX S6 – Statistical analyses for leaf chemistry comparisons between saplings and adults of the 12 common species**

### **Table S1. Normality tests for leaf chemistry comparisons between saplings and adults of the 12 common species.**

| **Clustering Type** | **Group/Species name** | **Stage** | **N** | ***w*** | ***P*** |
| --- | --- | --- | --- | --- | --- |
| Clustered species | Pool of clustered species | Saplings | 30 | 0.9637 | 0.47 |
|  |  | Adults | 30 | 0.9538 | 0.283 |
|  | *Acalypha cuneata* | Saplings | 5 | 0.9769 | 0.917 |
|  |  | Adults | 5 | 0.9375 | 0.648 |
|  | *Acidoton nicaraguensis* | Saplings | 5 | 0.9229 | 0.549 |
|  |  | Adults | 5 | 0.9439 | 0.693 |
|  | *Rinorea apiculata* | Saplings | 5 | 0.9861 | 0.964 |
|  |  | Adults | 5 | 0.8918 | 0.366 |
|  | *Rinorea viridifolia* | Saplings | 5 | 0.9312 | 0.604 |
|  |  | Adults | 5 | 0.9527 | 0.756 |
|  | *Macrolobium* 'yasuní' | Saplings | 5 | 0.9401 | 0.666 |
|  |  | Adults | 5 | 0.9517 | 0.749 |
|  | *Matisia oblongifolia* | Saplings | 5 | 0.8727 | 0.277 |
|  |  | Adults | 5 | 0.8412 | 0.168 |
| Non-clustered species | Pool of non-clustered species | Saplings | 30 | 0.9088 | 0.939 |
|  |  | Adults | 30 | 0.075 | 0.128 |
|  | *Pourouma bicolor* | Saplings | 5 | 0.842 | 0.1706 |
|  |  | Adults | 5 | 0.9761 | 0.9128 |
|  | *Sorocea steinbachii* | Saplings | 5 | 0.8788 | 0.304 |
|  |  | Adults | 5 | 0.9157 | 0.5024 |
|  | *Eugenia* 'minicomun' | Saplings | 5 | 0.9275 | 0.5797 |
|  |  | Adults | 5 | 0.7816 | 0.05689 |
|  | *Matisia malacocalyx* | Saplings | 5 | 0.929 | 0.5894 |
|  |  | Adults | 5 | 0.9075 | 0.4529 |
|  | *Neea* 'comun' | Saplings | 5 | 0.9427 | 0.6852 |
|  |  | Adults | 5 | 0.8281 | 0.1347 |
|  | *Eschweilera coriaceae* | Saplings | 5 | 0.9209 | 0.5355 |
|  |  | Adults | 5 | 0.9399 | 0.6653 |

N = number of sampled individuals, *w*= W-value, and *P= p*-value (where *P* ≤ 0.05 means significant difference to normal distribution ). Analyses were performed in PAST software v3.07 (Hammer *et al*., 2001).

### **Table S2.** ***t*- test table for chemical defence comparisons between saplings and adults of the 12 common species.**

### *t -* test analyses were performed in PAST statistical software v3.07 (Hammer *et al*., 2001). We assessed the equality of coefficient of variation (CV) of the population using the asymptotic test (Feltz & Miller, 1996; Krishnamoorthy & Lee, 2014). It was performed in R software using the library “cvequality” (R Development Core Team, 2017). *P*_AD = *p*-value of the asymptotic test.

| **Clustering Type** | **Group/Species** | **Stage** | **N** | **** | ***t*** | ***P*** | **% CV** | ***P*_AD** |
| --- | --- | --- | --- | --- | --- | --- | --- | --- |
| Clustered species | Pool of clustered species | Saplings | 30 | 20.63 | 0.654 | 0.515 | 17.79 | 0.814 |
|  |  | Adults | 30 | 20 |  |  | 19.12 |  |
|  | *Acalypha cuneata* | Saplings | 5 | 25.4 | -0.632 | 0.544 | 4.49 | 0.633 |
|  |  | Adults | 5 | 25.8 |  |  | 3.24 |  |
|  | *Acidoton nicaraguensis* | Saplings | 5 | 23.8 | 1.014 | 0.34 | 5.47 | 0.297 |
|  |  | Adults | 5 | 22.6 |  |  | 10.19 |  |
|  | *Rinorea apiculata* | Saplings | 5 | 21 | 1.087 | 0.309 | 8.91 | 0.256 |
|  |  | Adults | 5 | 19.6 |  |  | 11.18 |  |
|  | *Rinorea viridifolia* | Saplings | 5 | 20.2 | 0.404 | 0.697 | 5.42 | 0.645 |
|  |  | Adults | 5 | 19.8 |  |  | 9.71 |  |
|  | *Macrolobium* 'yasuní' | Saplings | 5 | 16.8 | 0.463 | 0.656 | 16.52 | **0.042** |
|  |  | Adults | 5 | 16.2 |  |  | 5.16 |  |
|  | *Matisia oblongifolia* | Saplings | 5 | 16.6 | 0.647 | 0.536 | 9.14 | 0.932 |
|  |  | Adults | 5 | 16 |  |  | 8.84 |  |
| Non-clustered species | Pool of non-clustered species | Saplings | 30 | 21.63 | -0.604 | 0.548 | 26.44 | 0.583 |
|  |  | Adults | 30 | 22.6 |  |  | 29.32 |  |
|  | *Pourouma bicolor* | Saplings | 5 | 20.8 | -3.959 | **0.004** | 5.27 | 0.843 |
|  |  | Adults | 5 | 23.6 |  |  | 4.83 |  |
|  | *Sorocea steinbachii* | Saplings | 5 | 19.8 | -0.962 | 0.364 | 6.59 | 0.411 |
|  |  | Adults | 5 | 20.8 |  |  | 9.25 |  |
|  | *Eugenia* 'minicomun' | Saplings | 5 | 30 | 0.189 | 0.854 | 6.67 | 0.126 |
|  |  | Adults | 5 | 29.6 |  |  | 14.45 |  |
|  | *Matisia malacocalyx* | Saplings | 5 | 17 | 0.802 | 0.445 | 7.2 | 0.913 |
|  |  | Adults | 5 | 16.4 |  |  | 6.95 |  |
|  | *Neea* 'comun' | Saplings | 5 | 14.6 | 0.132 | 0.544 | 6.13 | 0.664 |
|  |  | Adults | 5 | 14.2 |  |  | 7.71 |  |
|  | *Eschweilera coriaceae* | Saplings | 5 | 27.6 | -3.9 | **0.004** | 5.49 | 0.831 |
|  |  | Adults | 5 | 31 |  |  | 3.95 |  |

**References**

Feltz. C. J.. & Miller. G. E. (1996) An asymptotic test for the equality of coefficients of variation from k populations. *Statistics in Medicine*, 15(6), 647-658. [https:// 10.1002/(SICI)1097-0258(19960330)15:6<647::AID-SIM184>3.0.CO;2-P](https://w)

Hammer. Ř.. Harper. D.A.T.. Ryan. P.D. 2001. PAST: Paleontological statistics software package for education and data analysis, *Palaeontologia Electronica* 4(1): 9pp. <http://palaeo-electronica.org/2001_1/past/issue1_01.htm>.

Krishnamoorthy. K.. & Lee. M. (2014) Improved tests for the equality of normal coefficients of variation. *Computational Statistics*, 29(1-2), 215-232. <http://10.1007/s00180-013-0445-2>

# **APPENDIX S7 – Physical leaf trait comparisons between saplings and adult trees for each of the 12 common species**

**Table S1**. Average of leaf physical traits at intraspecific and ontogenetic level. N = number of sampled individuals,  = the average of the evaluated trait; *t* = the t value of the t-test; *P* = the significance value of the comparisons between saplings and adult trees for each evaluated trait. Bold *P* values are significant at *P* ≤ 0.01. The measured leaf traits were: thickness (mm). tearing (N_max_). punching (N_max_ × mm^-2^) and shearing (N × s × mm^-1^).

| **Clustered species** | | | | | | |  | **Non-clustered species** | | | | | | |
| --- | --- | --- | --- | --- | --- | --- | --- | --- | --- | --- | --- | --- | --- | --- |
| **Species** | **Physical Trait** | **Ontogenetic state** | **N** | **** | ***t*** | ***P*** |  | **Species** | **Physical Trait** | **Ontogenetic state** | **N** | **** | ***t*** | ***P*** |
| *Acalypha cuneata* | Thickness | sapling | 19 | 0.131 | -1.379 | 0.176 |  | *Pourouma bicolor* | Thickness | sapling | 10 | 0.286 | -9.143 | **< 0.001** |
|  |  | adult | 19 | 0.277 |  |  |  |  |  | adult | 20 | 0.719 |  |  |
|  | Tearing | sapling | 20 | 10.05 | -0.909 | 0.369 |  |  | Tearing | sapling | 10 | 17.544 | -6.201 | **< 0.001** |
|  |  | adult | 20 | 11.126 |  |  |  |  |  | adult | 19 | 37.622 |  |  |
|  | Punching | sapling | 18 | 0.277 | -0.886 | 0.382 |  |  | Punching | sapling | 10 | 0.257 | -0.922 | 0.364 |
|  |  | adult | 19 | 0.312 |  |  |  |  |  | adult | 20 | 0.294 |  |  |
|  | Shearing | sapling | 19 | 0.005 | -0.909 | 0.389 |  |  | Shearing | sapling | 10 | 0.019 | -3.044 | **0.005** |
|  |  | adult | 19 | 0.007 |  |  |  |  |  | adult | 20 | 0.034 |  |  |
| *Acidoton nicaraguensis* | Thickness | sapling | 19 | 0.131 | -1.809 | 0.078 |  | *Sorocea steinbachii* | Thickness | sapling | 18 | 0.168 | 1.873 | 0.069 |
|  |  | adult | 19 | 0.217 |  |  |  |  |  | adult | 18 | 0.125 |  |  |
|  | Tearing | sapling | 19 | 7.615 | 0.257 | 0.803 |  |  | Tearing | sapling | 19 | 9.987 | 1.701 | 0.090 |
|  |  | adult | 21 | 7.382 |  |  |  |  |  | adult | 19 | 12.856 |  |  |
|  | Punching | sapling | 19 | 0.166 | -1.594 | 0.149 |  |  | Punching | sapling | 19 | 0.242 | 0.172 | 0.864 |
|  |  | adult | 20 | 0.211 |  |  |  |  |  | adult | 19 | 0.233 |  |  |
|  | Shearing | sapling | 19 | 0.006 | 0.469 | 0.642 |  |  | Shearing | sapling | 19 | 0.01 | -1.841 | 0.073 |
|  |  | adult | 20 | 0.006 |  |  |  |  |  | adult | 19 | 0.012 |  |  |
| *Rinorea apiculata* | Thickness | sapling | 19 | 0.139 | 1.939 | 0.06 |  | *Eugenia* 'minicomun' | Thickness | sapling | 14 | 0.257 | 0.538 | 0.595 |
|  |  | adult | 19 | 0.127 |  |  |  |  |  | adult | 14 | 0.229 |  |  |
|  | Tearing | sapling | 20 | 9.794 | -1.586 | 0.121 |  |  | Tearing | sapling | 14 | 7.73 | 0.657 | 0.517 |
|  |  | adult | 20 | 11.305 |  |  |  |  |  | adult | 14 | 6.842 |  |  |
|  | Punching | sapling | 19 | 0.262 | -1.165 | 0.252 |  |  | Punching | sapling | 14 | 0.33 | 1.033 | 0.311 |
|  |  | adult | 19 | 0.309 |  |  |  |  |  | adult | 14 | 0.282 |  |  |
|  | Shearing | sapling | 20 | 0.006 | -0.643 | 0.524 |  |  | Shearing | sapling | 13 | 0.012 | 0.105 | 0.917 |
|  |  | adult | 20 | 0.007 |  |  |  |  |  | adult | 14 | 0.013 |  |  |
| *Rinorea viridifolia* | Thickness | sapling | 19 | 0.181 | 0.045 | 0.964 |  | *Matisia malacocalyx* | Thickness | sapling | 19 | 0.179 | 0.322 | 0.749 |
|  |  | adult | 18 | 0.179 |  |  |  |  |  | adult | 20 | 0.281 |  |  |
|  | Tearing | sapling | 20 | 8.228 | 0.677 | 0.502 |  |  | Tearing | sapling | 19 | 16.391 | 1.654 | 0.107 |
|  |  | adult | 20 | 7.708 |  |  |  |  |  | adult | 20 | 14.739 |  |  |
|  | Punching | sapling | 19 | 0.256 | 0.864 | 0.393 |  |  | Punching | sapling | 18 | 0.294 | 1.655 | 0.107 |
|  |  | adult | 20 | 0.228 |  |  |  |  |  | adult | 18 | 0.206 |  |  |
|  | Shearing | sapling | 20 | 0.006 | 0.115 | 0.909 |  |  | Shearing | sapling | 17 | 0.021 | -0.16 | 0.874 |
|  |  | adult | 20 | 0.006 |  |  |  |  |  | adult | 20 | 0.02 |  |  |
| *Macrolobium* 'yasuni' | Thickness | sapling | 20 | 0.161 | -1.403 | 0.169 |  | *Neea* 'comun' | Thickness | sapling | 20 | 0.231 | -0.991 | 0.328 |
|  |  | adult | 20 | 0.166 |  |  |  |  |  | adult | 20 | 0.241 |  |  |
|  | Tearing | sapling | 20 | 10.932 | -0.049 | 0.961 |  |  | Tearing | sapling | 19 | 11.691 | -0.23 | 0.819 |
|  |  | adult | 20 | 10.984 |  |  |  |  |  | adult | 20 | 11.949 |  |  |
|  | Punching | sapling | 20 | 0.281 | 1.473 | 0.149 |  |  | Punching | sapling | 19 | 0.272 | 0.752 | 0.457 |
|  |  | adult | 20 | 0.241 |  |  |  |  |  | adult | 19 | 0.244 |  |  |
|  | Shearing | sapling | 19 | 0.014 | 1.327 | 0.193 |  |  | Shearing | sapling | 20 | 0.012 | -2.017 | 0.051 |
|  |  | adult | 19 | 0.011 |  |  |  |  |  | adult | 20 | 0.016 |  |  |
| *Matisia oblongifolia* | Thickness | sapling | 19 | 0.152 | -1.821 | 0.076 |  | *Eschweilera coriacea* | Thickness | sapling | 20 | 0.155 | -0.459 | 0.649 |
|  |  | adult | 20 | 0.164 |  |  |  |  |  | adult | 20 | 0.167 |  |  |
|  | Tearing | sapling | 19 | 14.673 | -0.568 | 0.585 |  |  | Tearing | sapling | 19 | 13.787 | 0.007 | 0.994 |
|  |  | adult | 20 | 15.744 |  |  |  |  |  | adult | 20 | 13.779 |  |  |
|  | Punching | sapling | 19 | 0.162 | -2.991 | **0.005** |  |  | Punching | sapling | 20 | 0.297 | 1.263 | 0.214 |
|  |  | adult | 20 | 0.246 |  |  |  |  |  | adult | 20 | 0.257 |  |  |
|  | Shearing | sapling | 19 | 0.016 | 0.077 | 0.939 |  |  | Shearing | sapling | 20 | 0.021 | 1.088 | 0.283 |
|  |  | adult | 19 | 0.016 |  |  |  |  |  | adult | 20 | 0.019 |  |  |

# **APPENDIX S8 – Heatmap comparison of leaf resistance**

According to Boege and Marquis (2005), plants resistance traits may vary throughout their ontogeny. In order to assess how physical leaf traits vary throughout both ontogenetic stages and whether tree species present changing patterns of physical defence at intra– and interspecific level, we used *gplots* R package (R v3.0.0; R Development Core Team, 2013) to perform a heatmap and visualize the higher, lower and intermediate leaf resistance values from every sampled individual in the study.


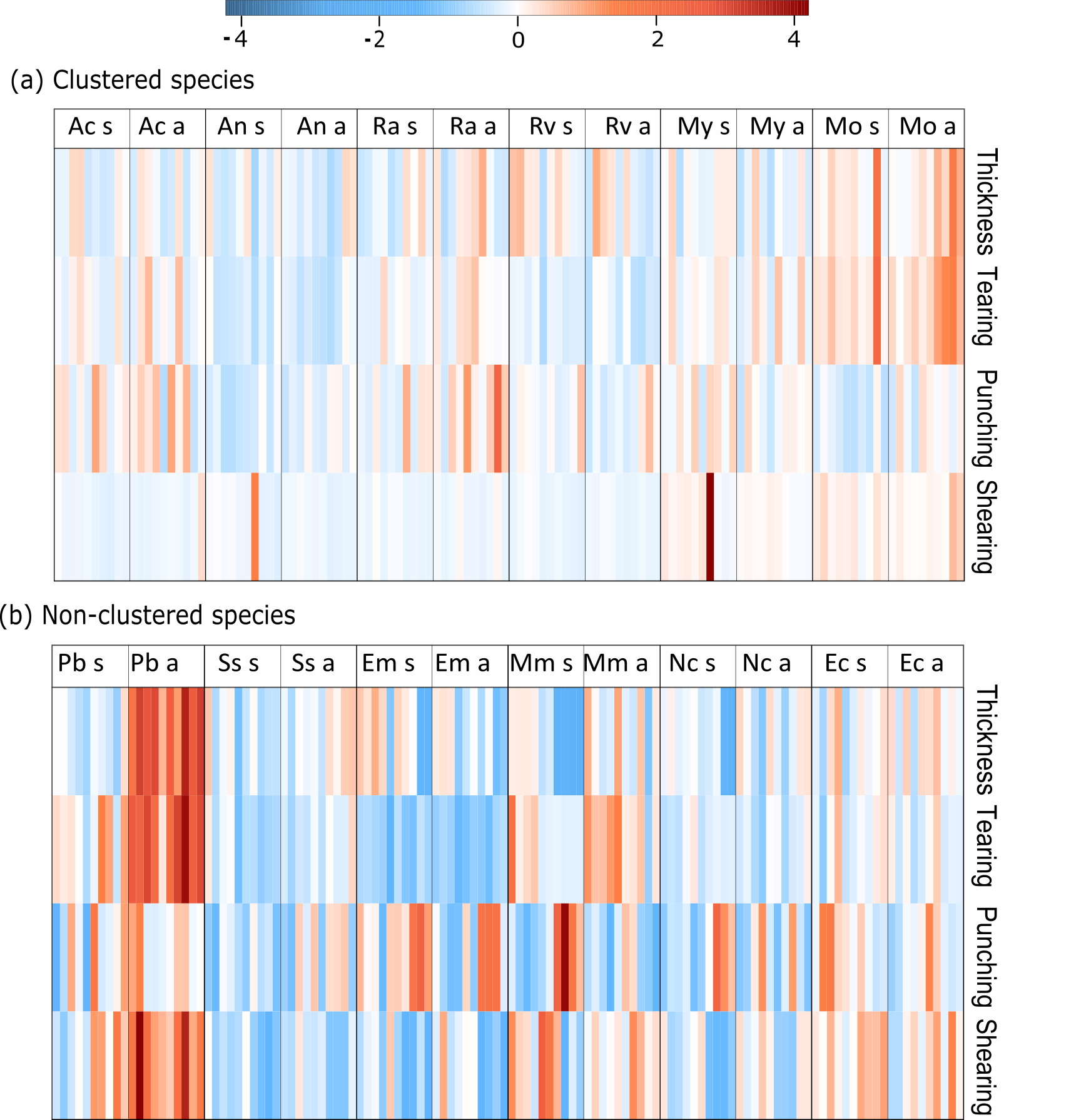


### **Figure S1.** Heatmaps comparison of physical leaf traits for (a) clustered species (b) non-clustered species. Intensity scale of leaf resistance values is represented by colours on the right side: dark blue denotes low resistance values; dark red denotes high resistance values and whitish colours denote intermediate resistance values. Each column represents every sampled individual, and each row is a physical trait. Saplings and adults are denoted by “s” and “a”. Species codes are detailed in Table 1.

**References**

Boege. K. & Marquis. R. J. (2005) Facing herbivory as you grow up: The ontogeny of resistance in plants. *Trends in Ecology and Evolution*, **20** (8), 441–448.

# **APPENDIX S9 – Principal component analysis (PCA) plot of chemical content for all species**

The similarities or differences in chemical compounds involving soluble metabolites of leaves between saplings and adult trees were assessed using a PCA performed in Metaboanalyst webserver 3.0 (Wiggins *et al*., 2016; Xia & Wishart, 2016). PCA for the pool of all species did not show dissimilarities between adults and saplings.


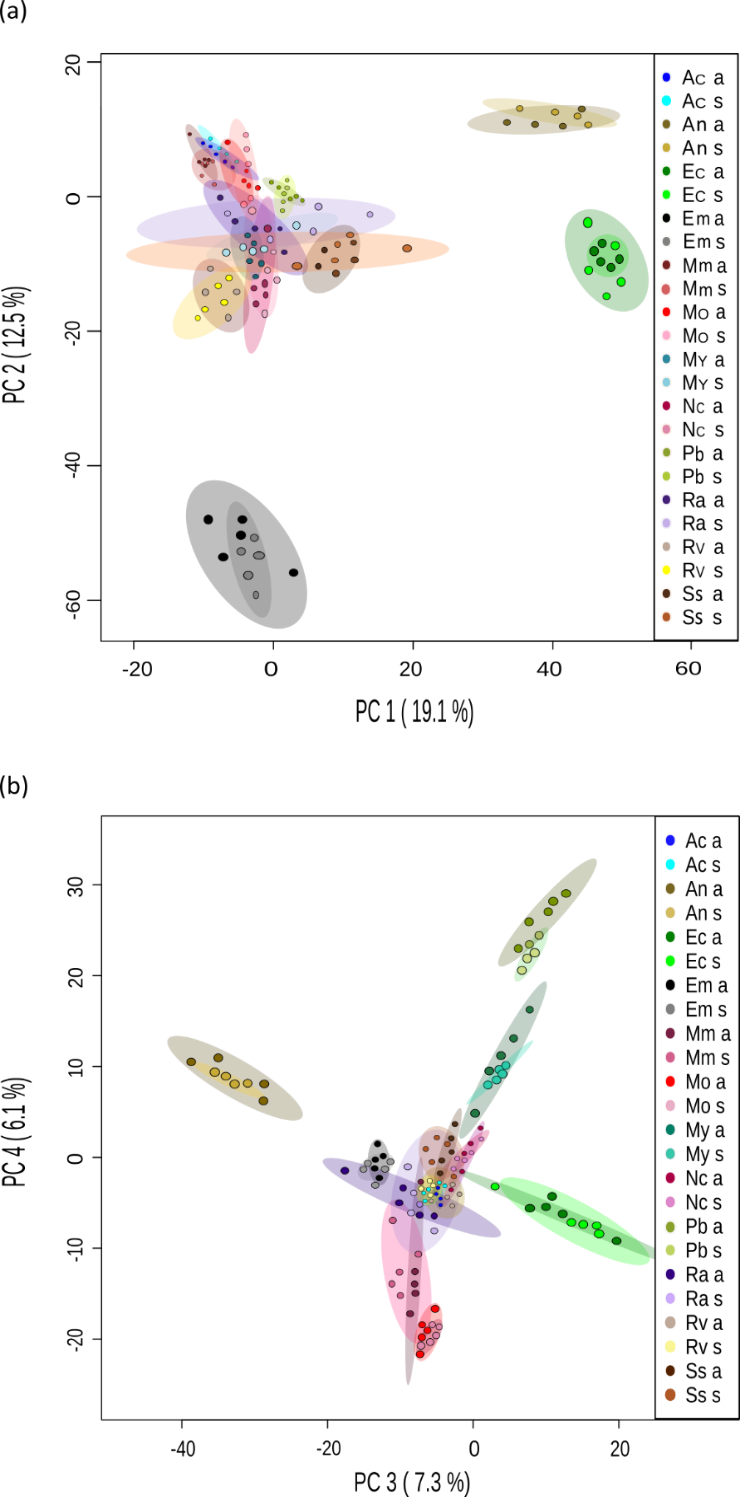


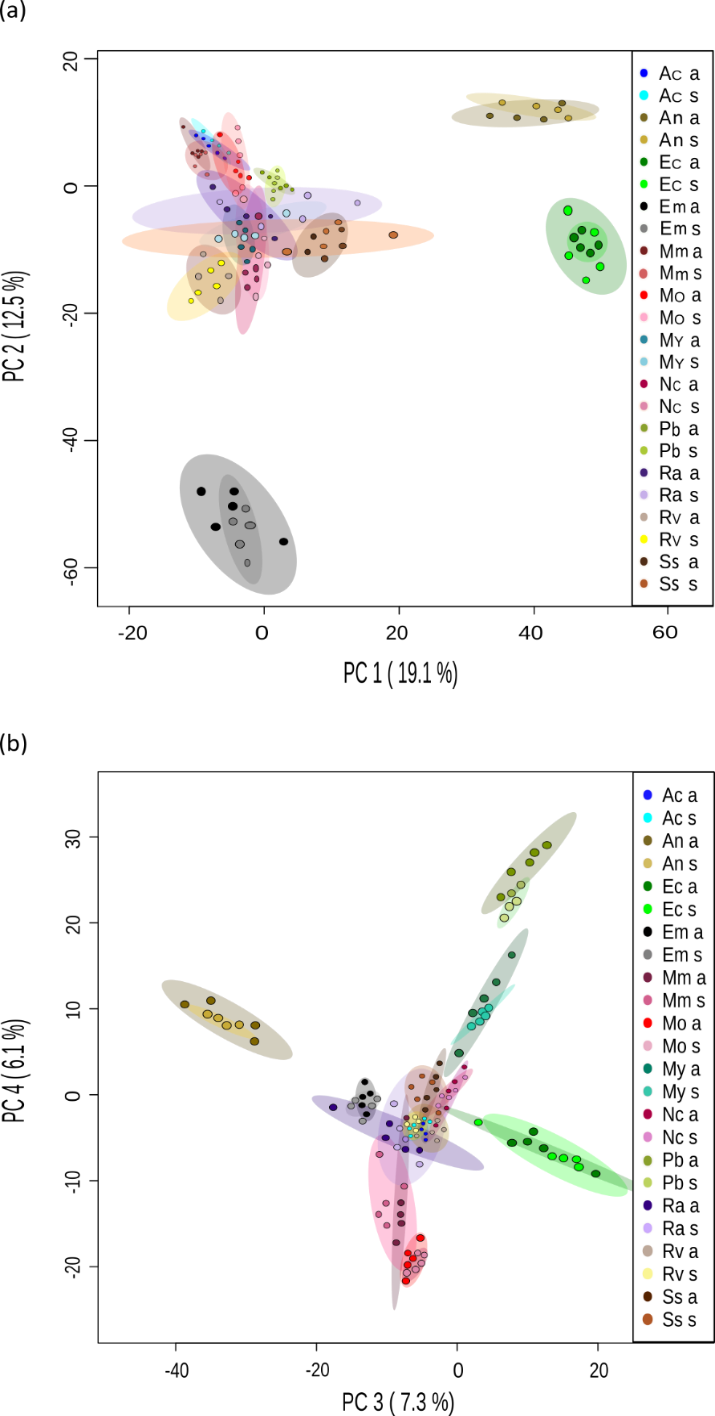


### **Figure S1.** Principal component analysis plot for all species, containing saplings and adults denoted by “s” and “a”, respectively. Values are derived from the total ion current of features obtained from UPLC-MS analysis. (a) First and second axes explained the 31.6% of variation. (b) Third and fourth axes explained the 13.4% of variation. The ellipses represent the 95% confidence regions. Species codes are detailed in Table 1.

**References**

Wiggins. N. L.. Forrister. D. L.. Endara. M. J.. Coley. P. D.. & Kursar. T. A. (2016) Quantitative and qualitative shifts in defensive metabolites define chemical defense investment during leaf development in Inga. a genus of tropical trees. *Ecology and Evolution*, 6(2), 478–492.

Xia. J & Wishart. D. 2016. Using MetaboAnalyst 3.0 for Comprehensive Metabolomics Data Analysis. *Current Protocols in Bioinformatics*. 55. 14.10.1-14.10.91.

# **APPENDIX S10 – Qualitative investment in chemical defences of saplings and adults from the pool of clustered and non-clustered species**


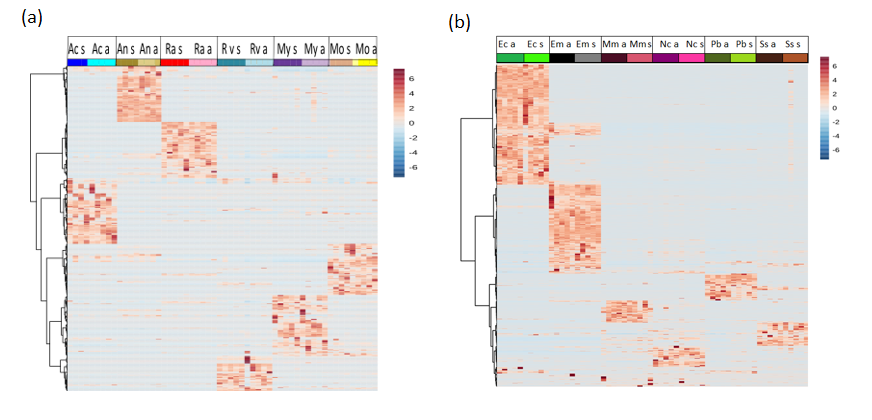


**
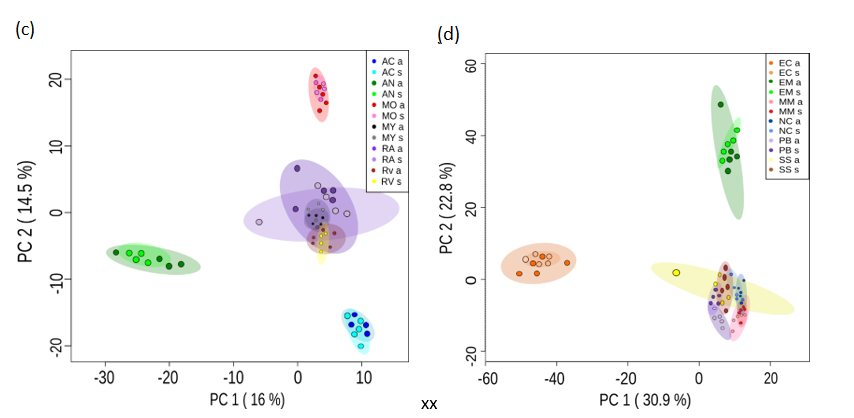

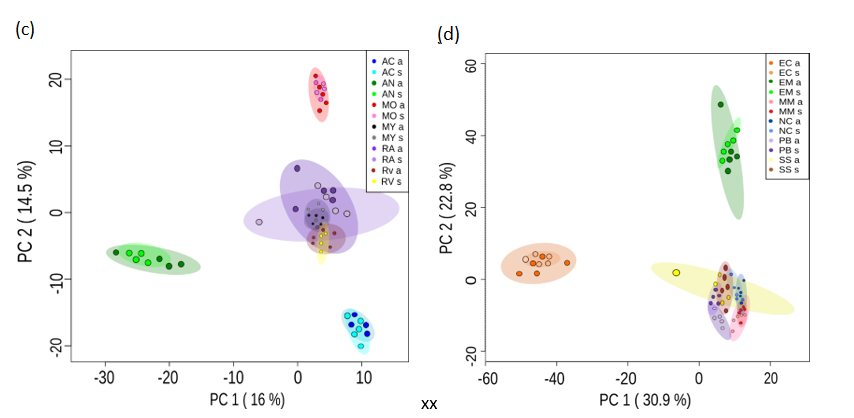
**

**Figure S1.** (a) Heatmap of metabolomic profile corresponding to the six clustered species. (b) Heatmap of metabolomic profile corresponding to the six non-clustered species. (c) PCA of soluble metabolites corresponding to the six clustered species. (d) PCA of soluble metabolites corresponding to the six non-clustered species.

# **APPENDIX S11 – Quantitative investment in chemical defences of saplings and adults from each of the 12 common species**

**a)** ***Acalypha cuneata***


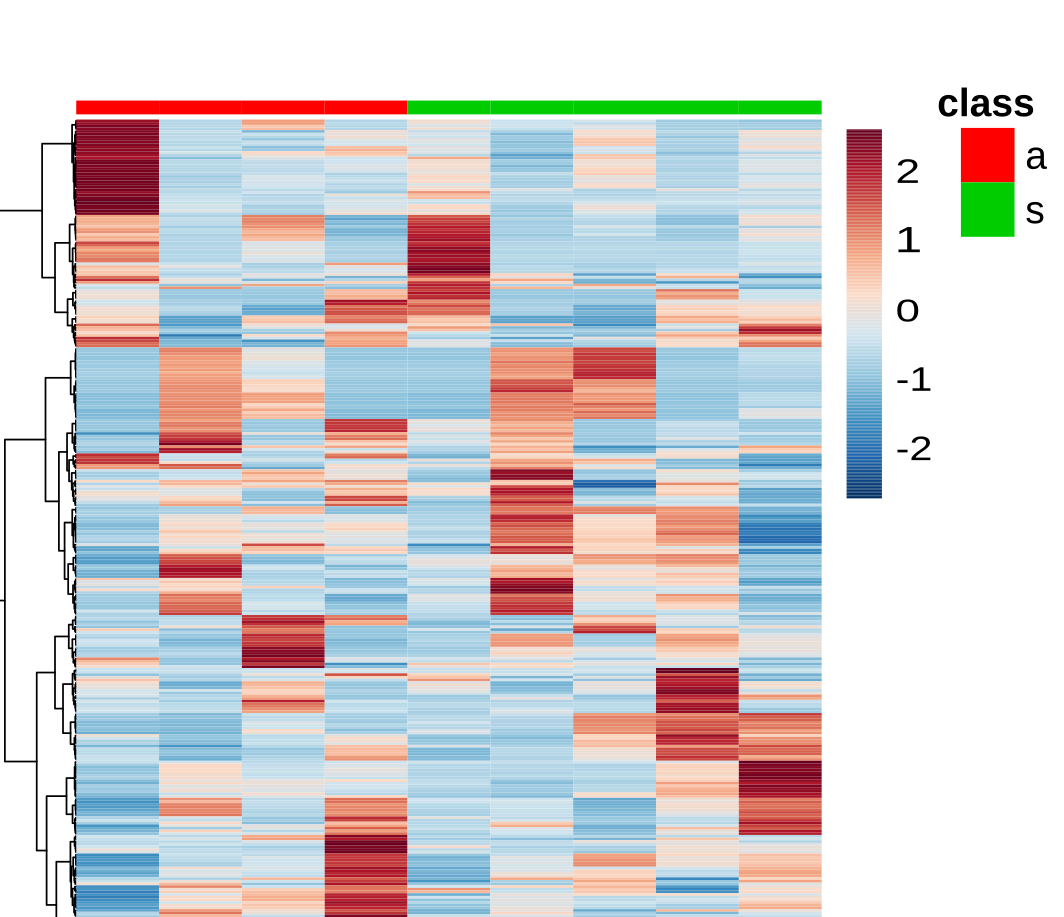

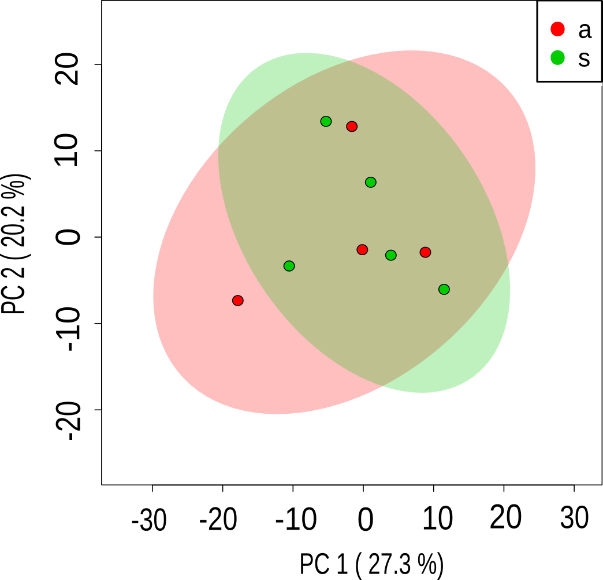


(i) Heatmap (ii) PCA

**b) *Acidoton nicaraguensis***


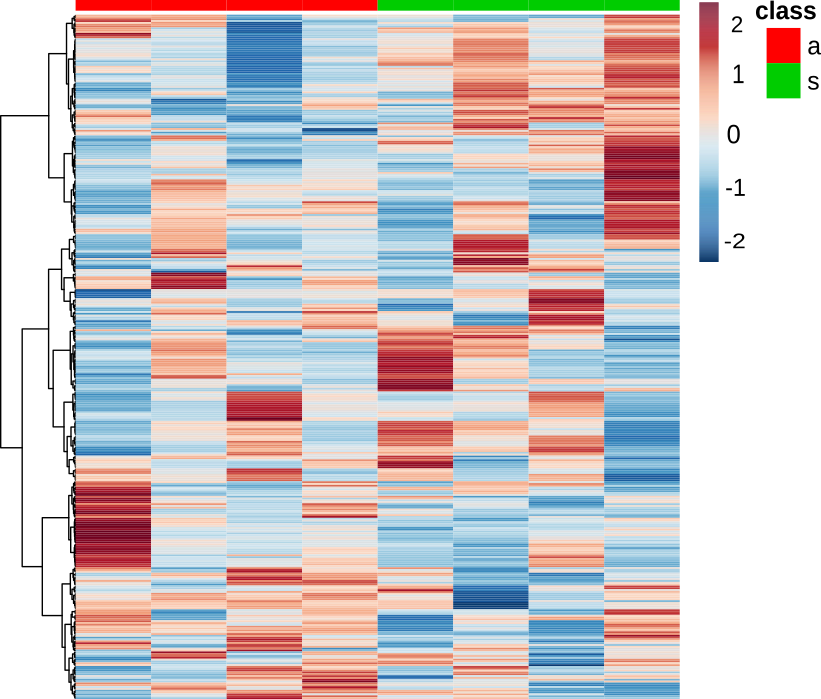

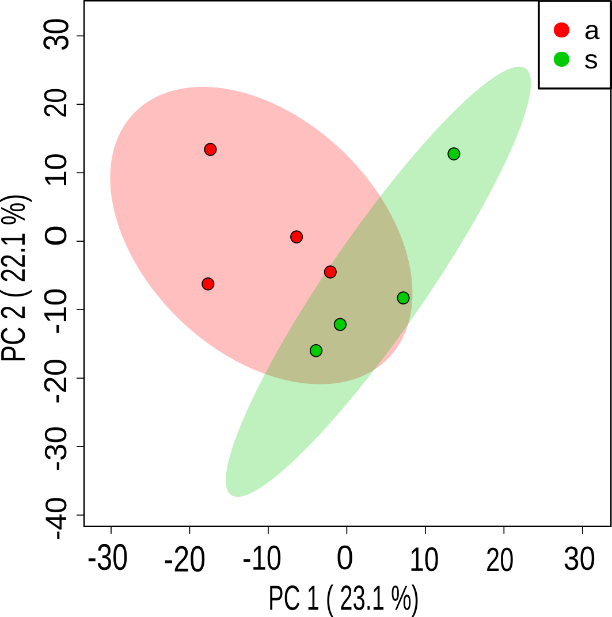


(i) Heatmap (ii) PCA

**c) *Rinorea apiculata***


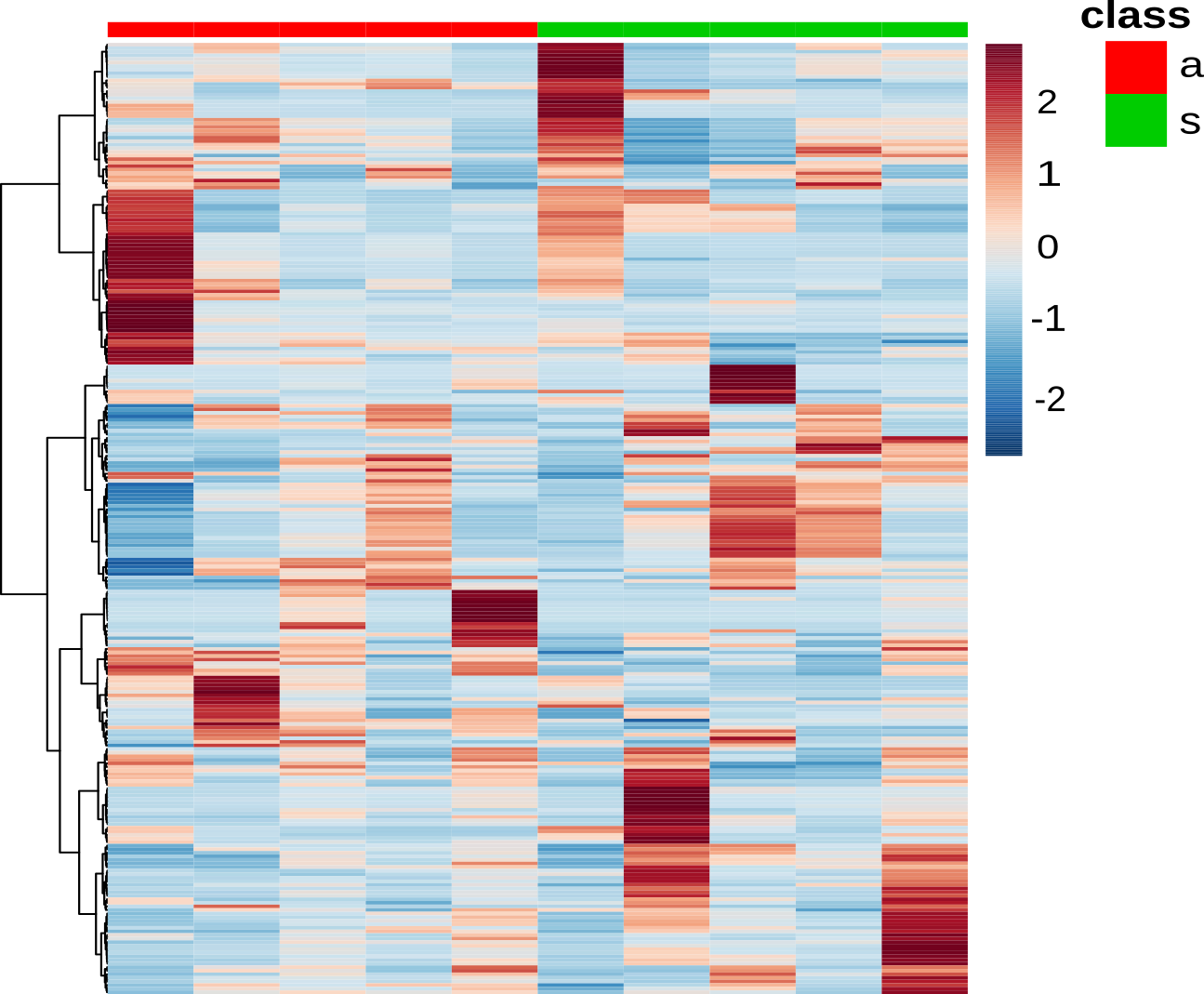

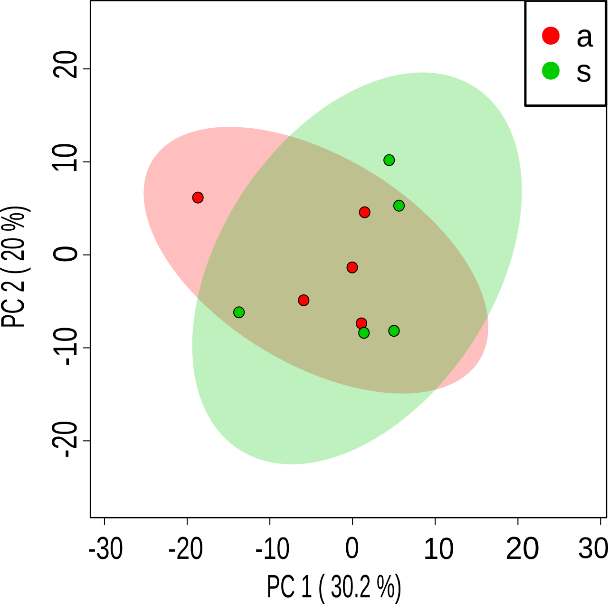


(i) Heatmap (ii) PCA

**d) *Rinorea viridifolia***


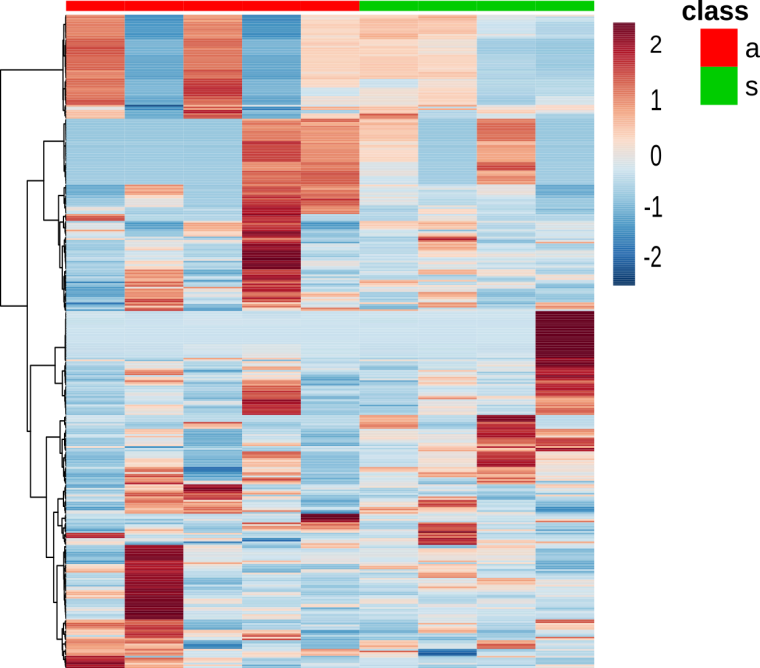

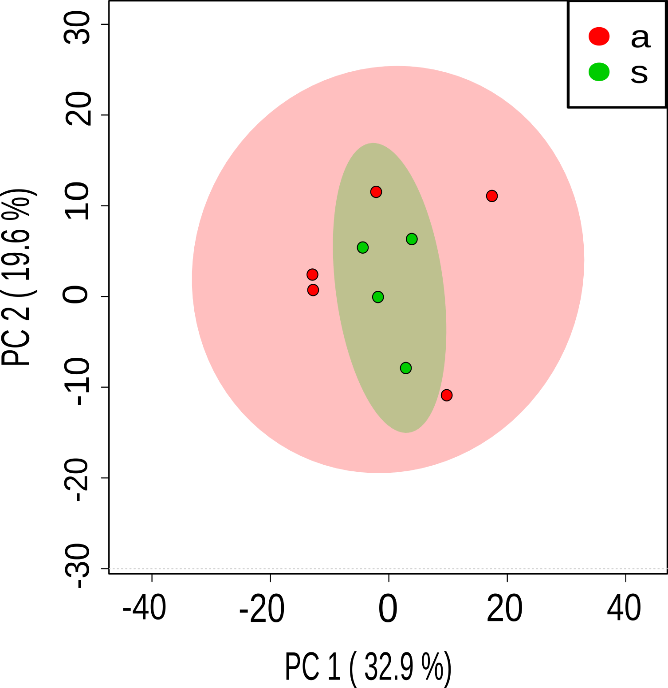


(i) Heatmap (ii) PCA

**(e) *Macrolobium* ‘yasuní’**


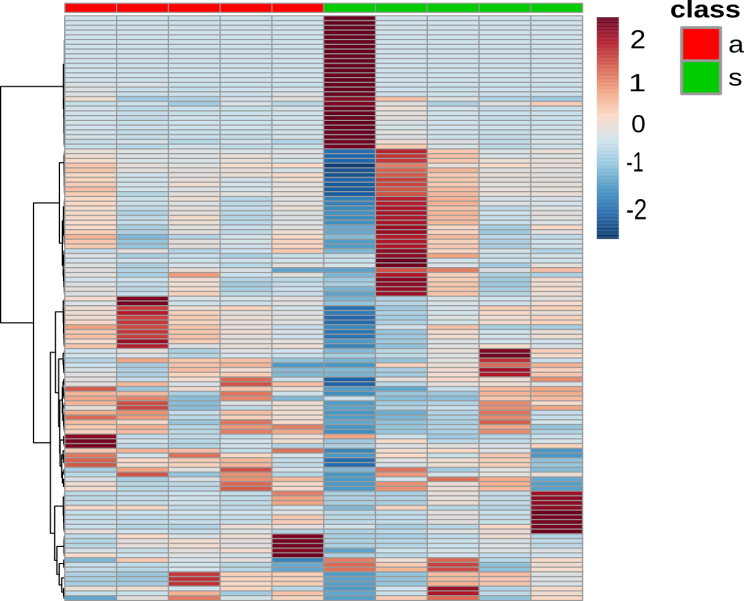

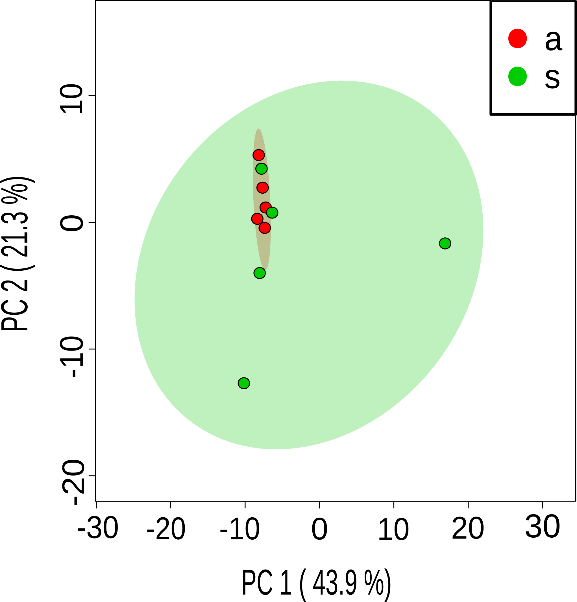


(i) Heatmap (ii) PCA

**f) *Matisia oblongifolia***


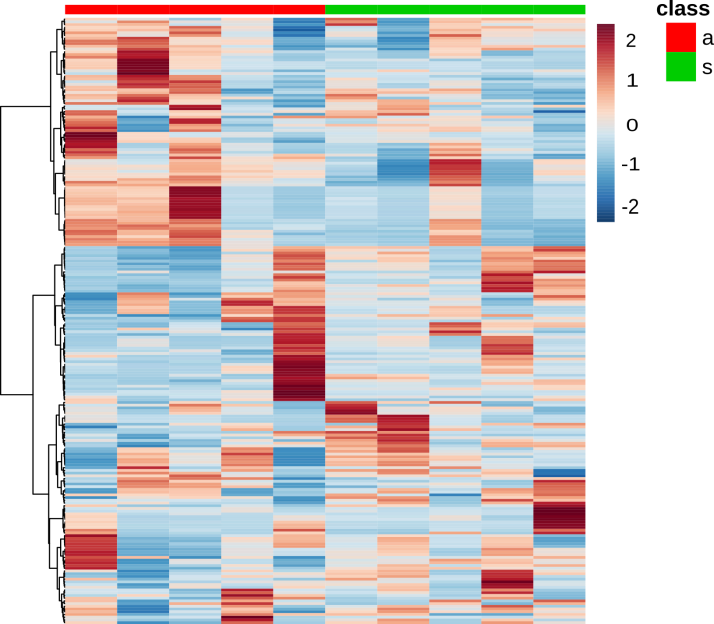

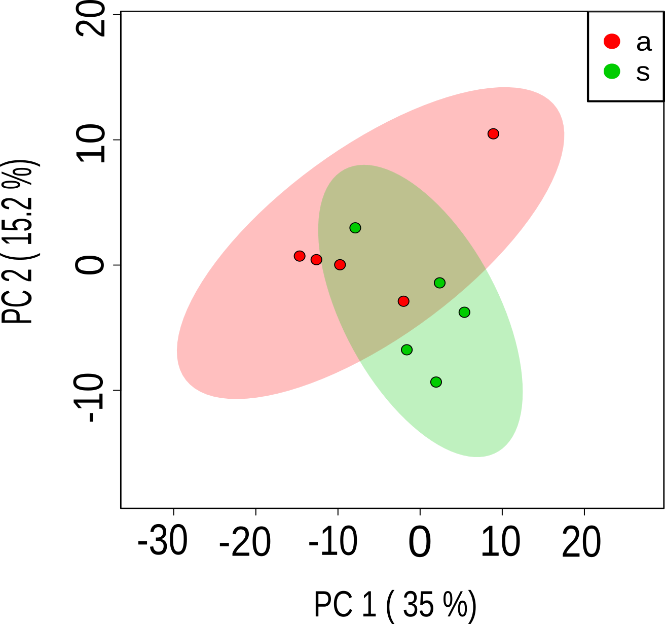


(i) Heatmap (ii) PCA

**g) *Eschweilera coriacea***

**
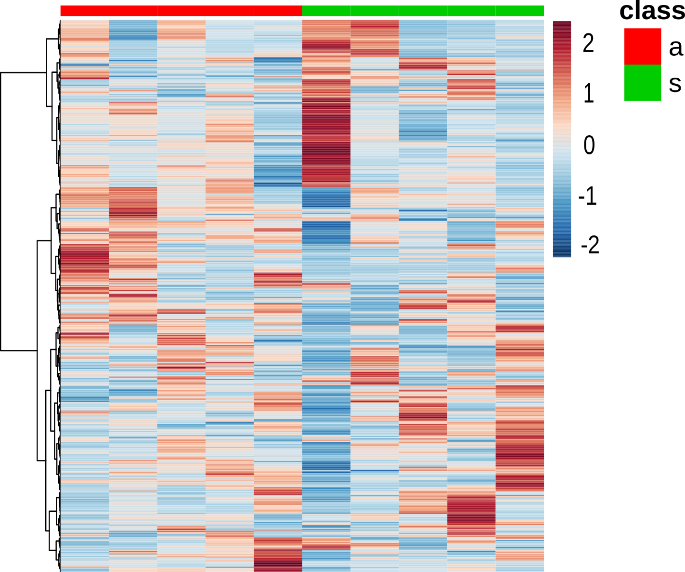

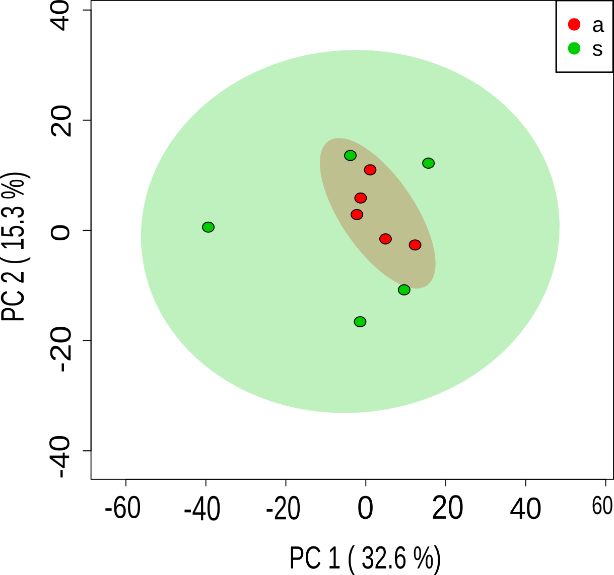
**

(i) Heatmap (ii) PCA

**h) *Eugenia* ´minicomun’**


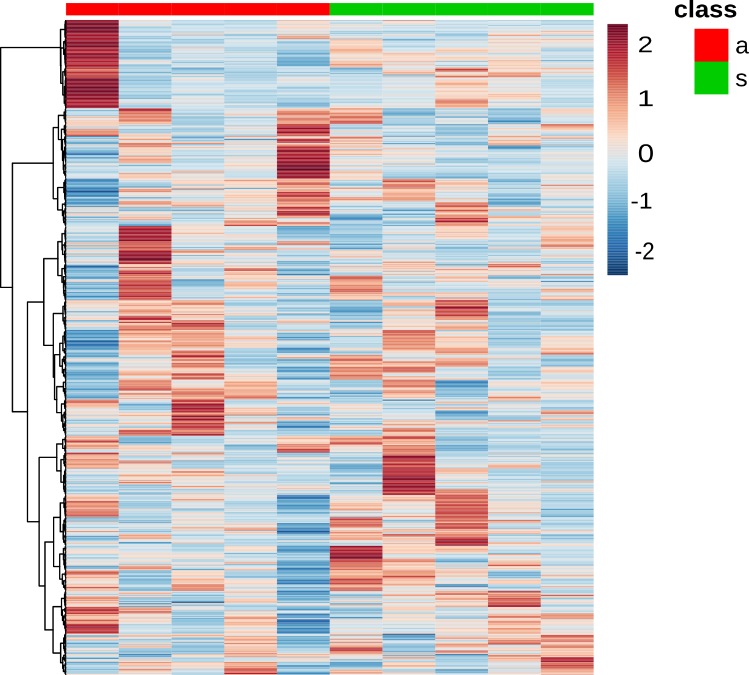

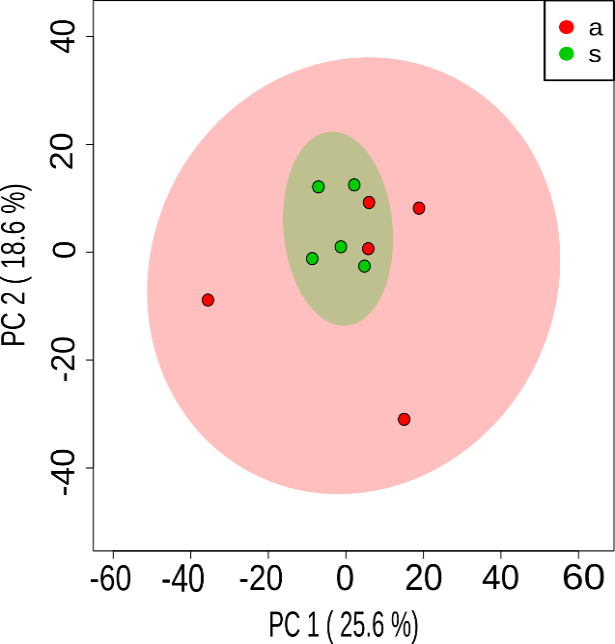


(i) Heatmap (ii) PCA

**i) *Matisia malacocalyx***


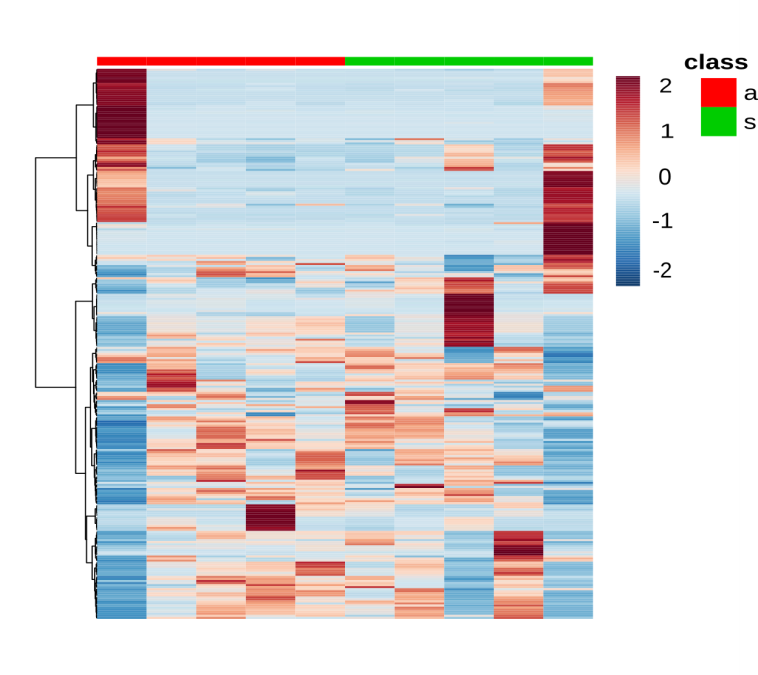

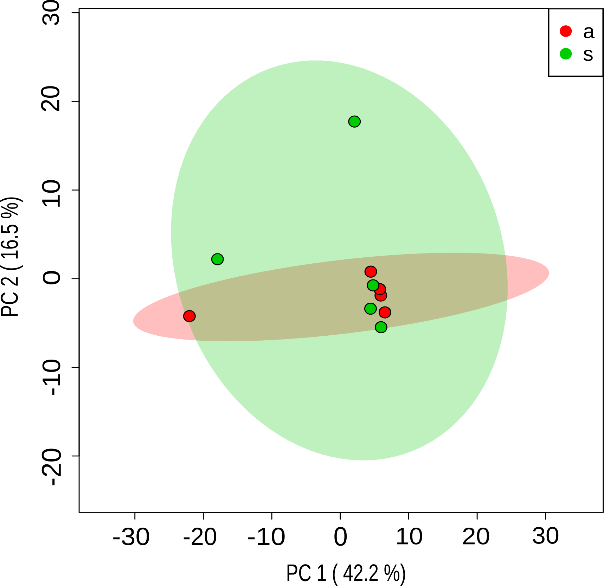


(i) Heatmap (ii) PCA

**j) *Neea* ‘comun’**

**
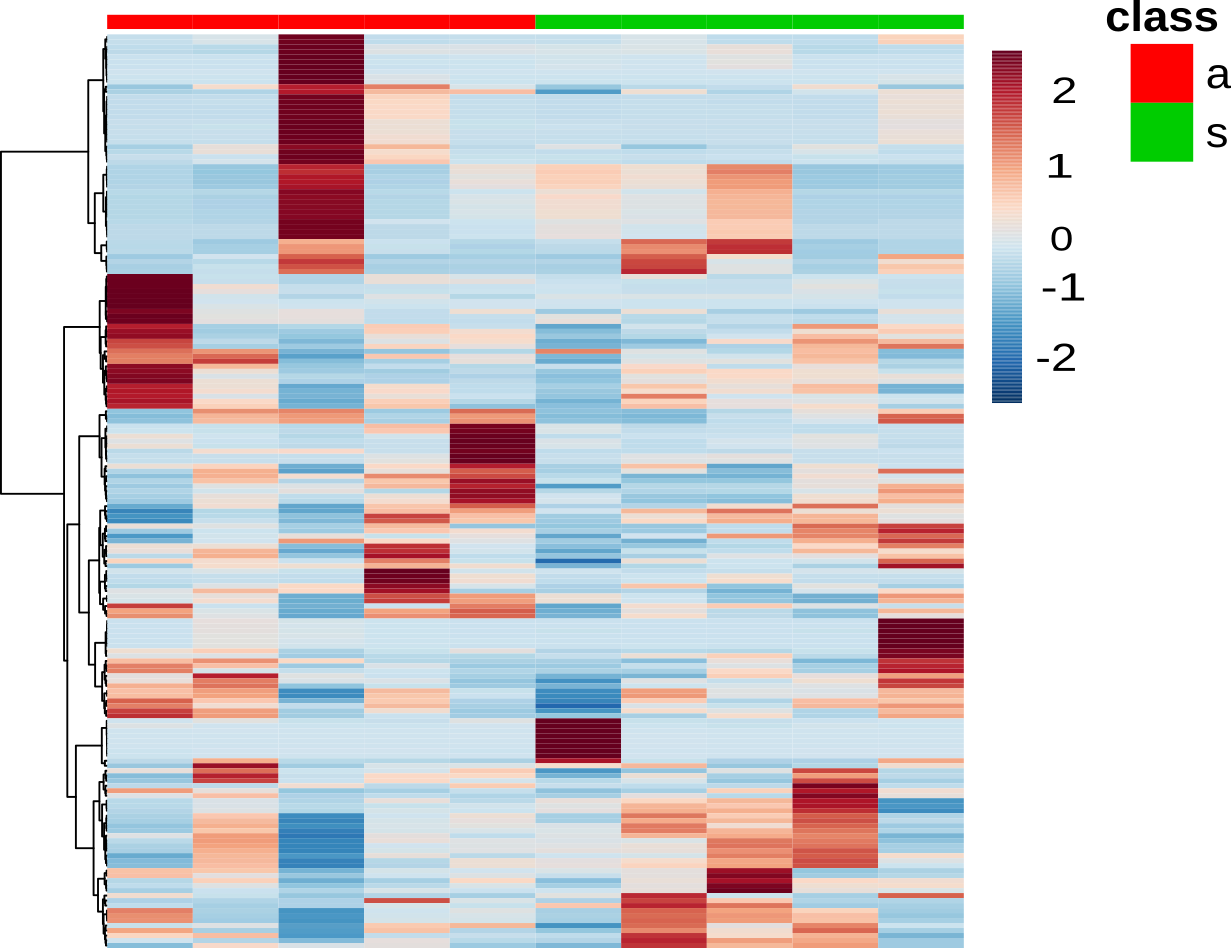

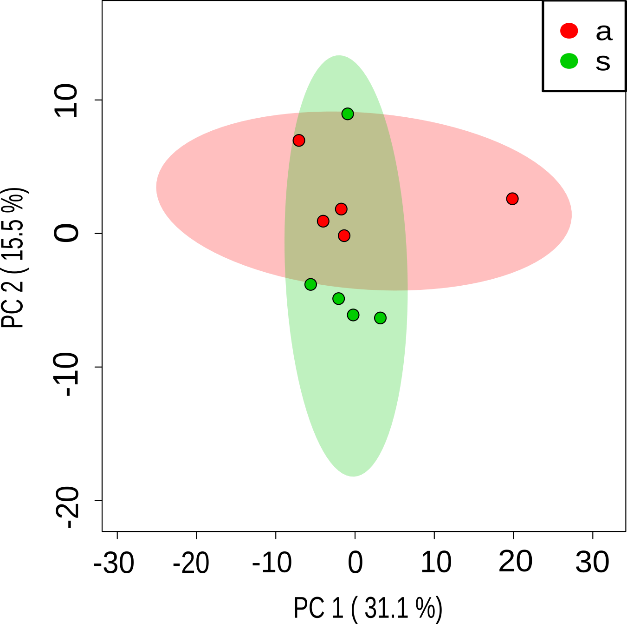
**

(i) Heatmap (ii) PCA

**k) *Pourouma bicolor***


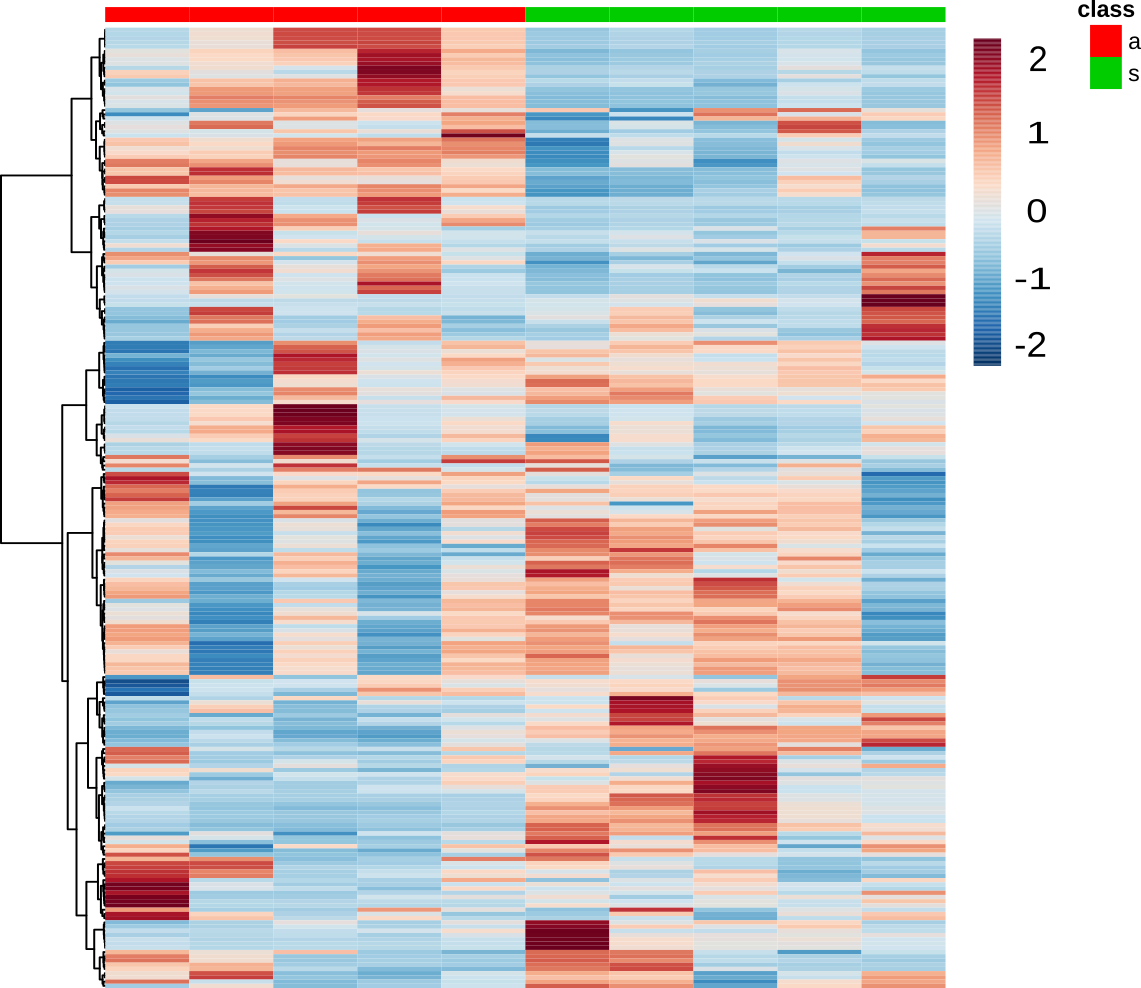

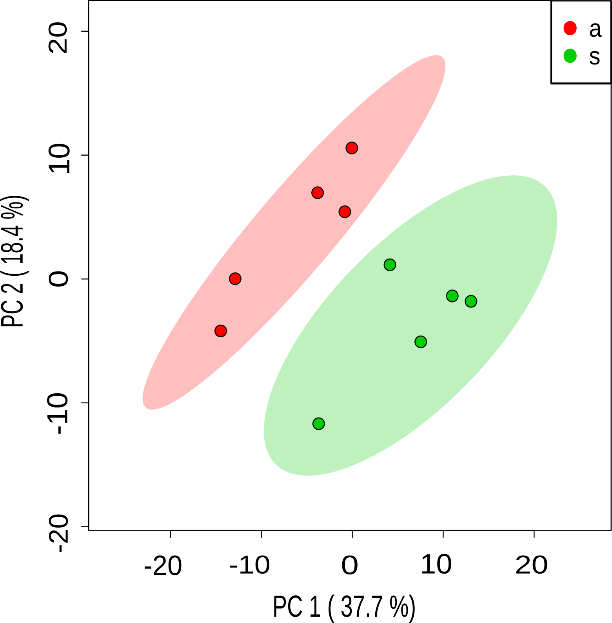


(i) Heatmap (ii) PCA


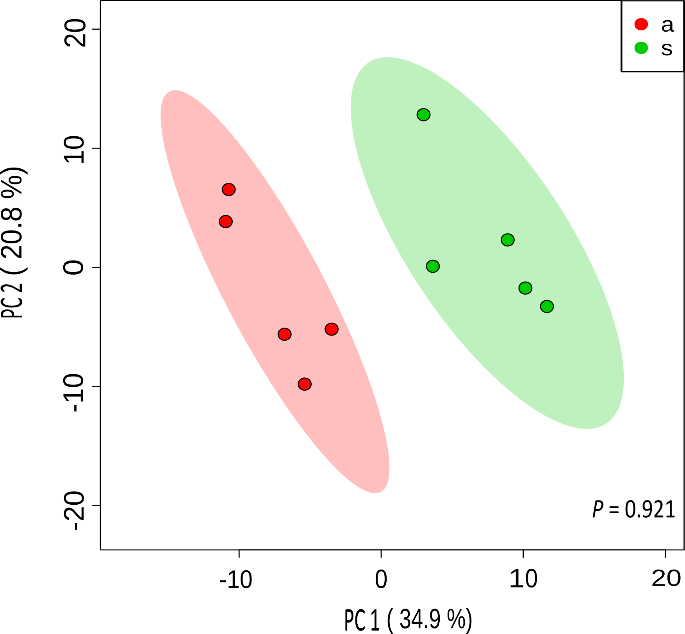


(iii) PLS-DA

The *P* value based on permutation test of

separation distance shows samples did not differ

in their metabolomics profile.

**l) *Sorocea steinbachii***


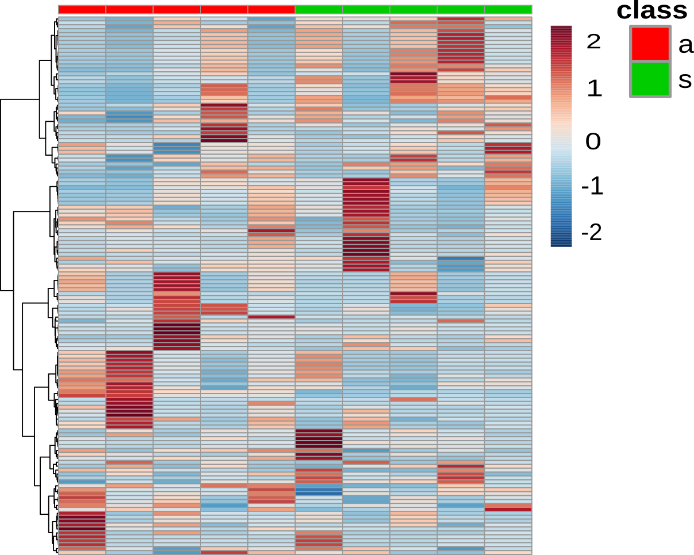

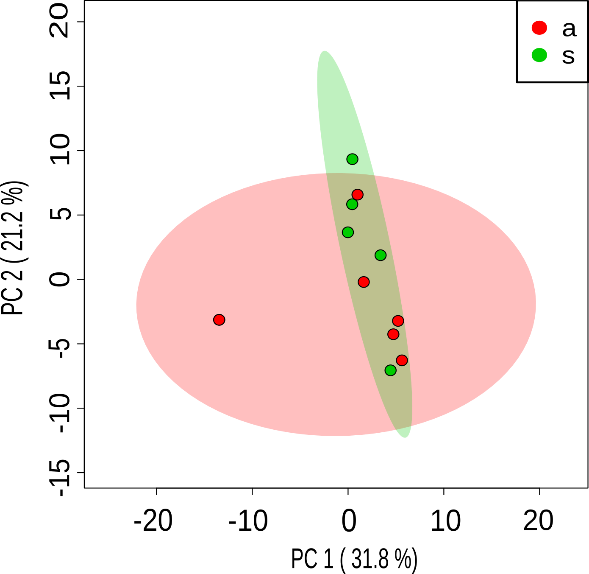


(i) Heatmap (ii) PCA

# **APPENDIX S12 – Relationships between spatial clustering and physical leaf traits variability**

**
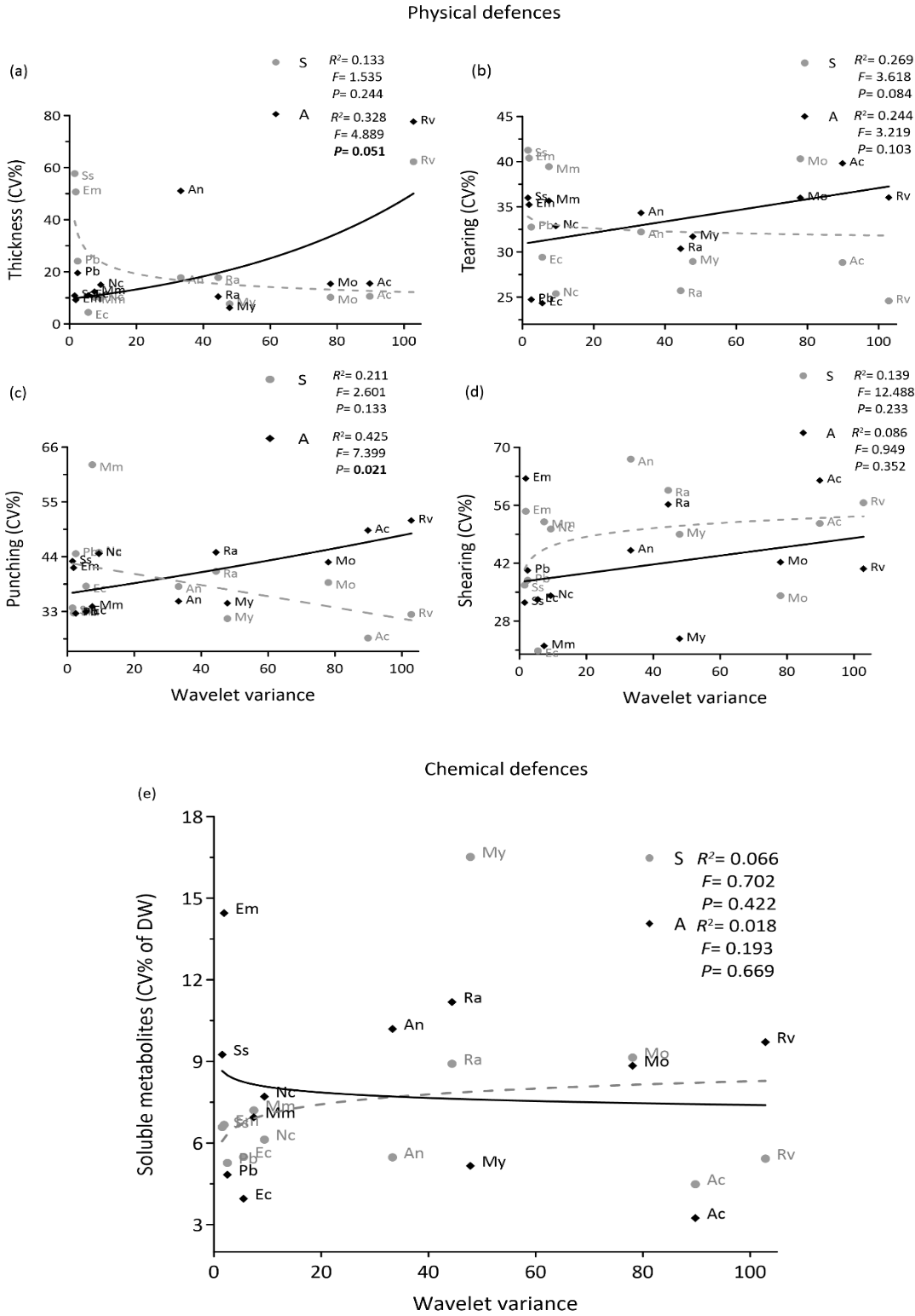
**

# **Figure S1.** Relationships between spatial clustering (wavelet variance, X axis) and physico-chemical leaf traits variability (Y axis). Higher values of wavelet variance represent clustered species of our 12 focal common tree species in Yasuní National Park. Gray dots represent saplings. Black diamonds represent adults. Solid and dashed lines represent the regression of the relationship between wavelet variance and the variation of each physico-chemical defences. S = saplings; A = adults. *P*, *F* and *R*^2^ are ANOVA statistical values from each fitted equation model: (a) *y*_S_ = 44.373*x*^-0.278^, *y*_A_ = 9.613 × 62.311*^x^*; (b) *y*_S_ = 38.513*x*^-0.065^, *y*_A_ = 30.899 + 0.062*x*; (c) *y*_S_ = 41.798 – 0.112*x*, *y*_A_ = 36.602 × -360.993*^x^* and (d) *y*_S_ = 39.286 + 3.034ln*x*, *y*_A_ = 37.447 + 0.106*x* and (e) *y*_S_ = 5.846 + 0.527ln*x*, *y*_A_ = 8.787*x*^-0.037^. Species codes are detailed in Table 1.

# **APPENDIX S13 – Relationship between physical leaf trait and level of spatial clustering**

**
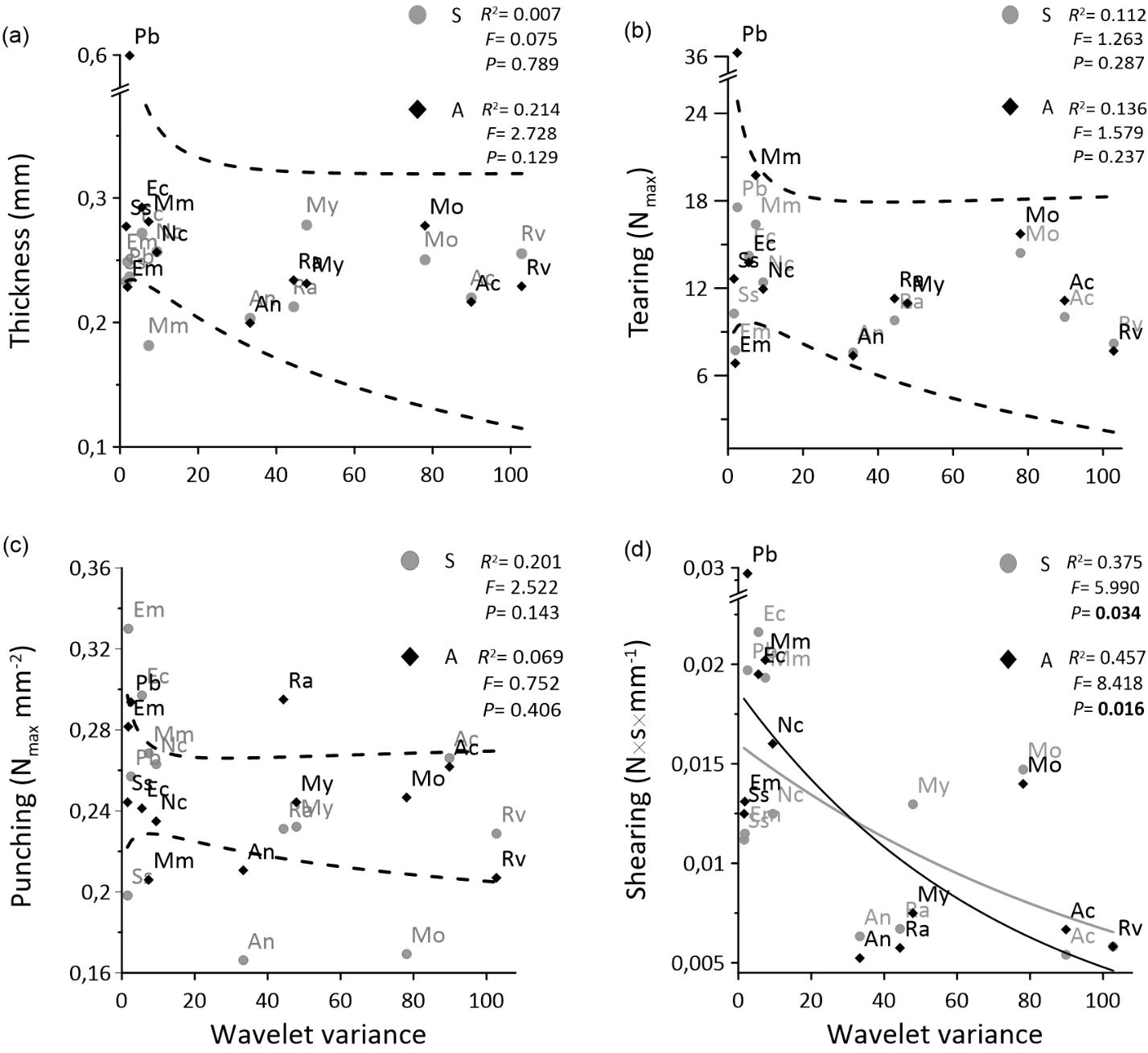
**

**Figure S1**. Relationships between physical leaf traits (Y axis) and wavelet variance (*X* axis; level of spatial clustering) of our 12 focal common tree species in Yasuní National Park. Gray dots reprent saplings. Black diamonds represent adults. Solid lines represent the regression of the relationship between wavelet variance and force to shear. Upper and lower dashed lines represent 5% and 95% confidence intervals of non-significant regressions. S = saplings; A = adults. *P*. *F* and *R*^2^ are ANOVA statistical values from each fitted equation model: (a) *y*_S_ = 0.235 + 6.65e^-05^*x*. *y*_A_ = 0.361-0.031ln*x*; (b) *y*_S_ = 12.704 – 0.030*x*. *y*_A_ = 18.856 – 1.867ln*x*; (c) *y*_S_ = 0.279 -0.014ln*x*. *y*_A_ = 0.261*x* ^-0.021^ and (d) *y*_S_ = 0.016 ×144.93*^x^*. *y*_A_= 0.0187 × 73.483*^x^*. Species codes are detailed in Table 1.

# **APPENDIX S14 – Averages of the diameter at breast height (dbh) of sampled trees**

Stem diameter at the breast height (dbh) of every sampled individual tree was recorded at 1.3 m above the ground and measured under two criteria. For trees ≤ 30 mm in stem diameter, we used a digital caliper (Fowler Tools of Canada, Ontario, Canada) while for trees > 30 mm in stem diameter a diameter tape was used (Forestry suppliers, Inc, Metric Fabric Diameter Tape Model 283D/5M, Mississippi, USA).

Based on census data carried out in YFDP during periods: 1995-2000. 2002-2006. 2007-2008 (R. Valencia. *unpublished data*) and 2015 (personal measurements), we calculated the growth rate (GR) and the relative growth rate (RGR) of the studied species estimated by the following equation (Hoffman *et al*., 2002):

RGR = $\frac{(lnW_{2}- \ln W_{1})}{(t_{2} - t_{1})}$

where *W_1_* is the stem diameter at time one (*t_1_*) and *W_2_* is the stem diameter at time two (*t_2_*). Time data were calculated, considering the exact number of days between the dates of the first and the second census and then processed as an annual rate (mm × year^-1^). We estimated the RGR because this equation considers light availability, size and age of trees as limiting factors for plant growth (Boyden *et al*., 2009; Philipson *et al*., 2011).

### **Table S1. Diameter at breast height of our tree focal species including averages for saplings and adults**. The table shows the number of sampled individuals (N), growth rate (GR) in mm, relative growth rate (RGR) in mm × year^-1^ and the type of habitat they inhabit (based on Valencia *et al*., 2004a topographic classification). The RGR average of saplings of non-clustered species was 0.034 ± 0.015 mm × year^-1^; for adults of non-clustered species was 0.016 ± 0.004 mm × year^-1^; for saplings of clustered species was 0.022 ± 0.003 mm × year^-1^; for adults of clustered species was 0.017 ± 0.006 mm × year^-1^.

| Species name | Stage | N | GR | RGR | Cluster status | Habitat |
| --- | --- | --- | --- | --- | --- | --- |
| *Acalypha cuneata* | sapling | 10 | 0.31 | 0.024 | clustered | valley |
|  | adult | 10 | 0.50 | 0.014 |  |  |
| *Acidoton nicaraguensis* | sapling | 10 | 0.19 | 0.017 | clustered | ridge |
|  | adult | 10 | 0.31 | 0.026 |  |  |
| *Rinorea viridifolia* | sapling | 10 | 0.33 | 0.022 | clustered | valley |
|  | adult | 10 | 0.57 | 0.013 |  |  |
| *Rinorea apiculata* | sapling | 10 | 0.43 | 0.022 | clustered | ridge |
|  | adult | 10 | 1.28 | 0.023 |  |  |
| *Macrolobium* 'yasuní' | sapling | 10 | 0.32 | 0.025 | clustered | ridge |
|  | adult | 10 | 0.75 | 0.013 |  |  |
| *Matisia oblongifolia* | sapling | 10 | 0.28 | 0.026 | clustered | ridge |
|  | adult | 10 | 0.33 | 0.012 |  |  |
| *Pourouma bicolor* | sapling | 10 | 2.63 | 0.054 | non-clustered | ridge |
|  | adult | 10 | 11.07 | 0.015 |  |  |
| *Sorocea steinbachii* | sapling | 10 | 0.58 | 0.047 | non-clustered | ridge |
|  | adult | 10 | 1.25 | 0.022 |  |  |
| *Eugenia* 'minicomun' | sapling | 10 | 0.21 | 0.014 | non-clustered | ridge |
|  | adult | 10 | 0.93 | 0.022 |  |  |
| *Matisia malacocalyx* | sapling | 10 | 0.49 | 0.024 | non-clustered | ridge |
|  | adult | 10 | 0.78 | 0.012 |  |  |
| *Neea* 'comun' | sapling | 10 | 0.61 | 0.036 | non-clustered | ridge |
|  | adult | 10 | 0.86 | 0.014 |  |  |
| *Eschweilera coriacea* | sapling | 10 | 0.76 | 0.029 | non-clustered | ridge |
|  | adult | 10 | 3.18 | 0.013 |  |  |

**References**

Boyden. S. B.. Reich. P. B.. Puettmann. K. J.. & Baker. T. R. (2009) Effects of density and ontogeny on size and growth ranks of three competing tree species. *Journal of Ecology*, 97(2), 277–288.

Hoffmann. W. A. (2002) Avoiding Bias in Calculations of Relative Growth Rate. *Annals of Botany*, 90. 37–42.

Philipson. C. D.. Saner. P.. Marthews. T.. Nilus. R.. Reynolds. G.. Turnbull. L. & Hector. A. (2011) Light-based Regeneration Niches: Evidence from 21 Dipterocarp Species using Size-specific RGRs. *Biotropica,* 44. 627–636.

Valencia. R.. Foster. R.B.. Villa. G.. Condit. R.G.. Svenning. J.C.. Hernandez. C.. Romoleroux. K.. Losos. E.C.. Magard. E. & Balslev. H. (2004a) Tree species distributions and local habitat variation in the Amazon: large forest plot in eastern Ecuador. *Journal of Ecology*, 92, 214–229.
